# A Review on the Application of Agri-Food Residues as Biosorbents of Heavy Metal Ions

**DOI:** 10.3390/ma19204414

**Published:** 2026-10-20

**Authors:** Kamila Koronkiewicz, Monika Kalinowska, Grzegorz Świderski, Krzysztof Jan Kurzydłowski, Tomasz Płociński, Rafat Al Afif

**Affiliations:** 1 Department of Chemistry, Biology and Biotechnology, Institute of Environmental Engineering and Energetics, Faculty of Civil Engineering and Environmental Sciences, Białystok University of Technology, Wiejska 45E Street, 15-351 Białystok, Poland; m.kalinowska@pb.edu.pl (M.K.); g.swiderski@pb.edu.pl (G.Ś.); 2 Department of Material and Production Engineering, Institute of Mechanical Engineering, Faculty of Mechanical Engineering, Białystok University of Technology, Wiejska 45C Street, 15-351 Białystok, Poland; k.kurzydlowski@pb.edu.pl; 3 Faculty of Materials Science and Engineering, Warsaw University of Technology, Wołoska 141 Street, 02-507 Warszawa, Poland; tomasz.plocinski@pw.edu.pl; 4 Institute of Chemical and Energy Engineering, University of Natural Resources and Life Sciences, Muthgasse 107, 1190 Vienna, Austria; rafat.alafif@boku.ac.at

**Keywords:** adsorption, agri-food waste, metal ions, water, wastewater

## Abstract

Surface water contamination is widespread worldwide, including contamination caused by heavy metal cations. These pollutants originate from large volumes of effluents from various industries, agriculture, and municipal sources. Therefore, in recent years, it has become necessary to search for innovative, cheap and efficient techniques to remove these pollutants. This review presents adsorption as the most promising compared with alternatives, such as membrane filtration and electrochemical oxidation. It lists examples of the use of agri-food industry wastes and their chemical, biological and thermal modifications, along with their performance against individual metal ions in aqueous solutions. In addition, various techniques necessary to test the sorption potential of adsorbents are described. Based on the literature, it can be seen that adsorption using plant waste materials is a simple and cost-effective technique that is also in line with the principles of a closed-loop system.

## 1. Introduction

Currently, the environment is exposed to various harmful factors resulting from pollution. There is a risk of accumulation of toxic residues, which are disrupting the balance of the ecosystem. The main sources of heavy metal contamination in wastewater and aquatic environments are agriculture (fertilizers, plant incineration), industry (batteries, paint manufacturing), and the transportation sector. Some pollutants originate from natural sources, such as the weathering of rocks or volcanic eruptions. Considerable attention is devoted to the study of metal-ion pollution [1,2,3]. There are many systems for treating wastewater and water contaminated with heavy metal ions, including ion exchange, membrane filtration and coagulation. Among the available treatment technologies, membrane processes such as nanofiltration can achieve very high heavy metal removal efficiency. Their practical application is limited by membrane fouling and relatively high energy consumption [4,5,6]. Chemical precipitation and coagulation-flocculation are widely applied in industry, but their efficiency decreases at low metal-ion concentrations and substantial amounts of sludge are generated, increasing disposal costs. Electrochemical methods (e.g., electrocoagulation) have also demonstrated high removal efficiencies for various inorganic contaminants [6,7], but their implementation is frequently associated with elevated energy demand and electrode consumption. Ion-exchange processes are highly selective and effective, but the regeneration of resins requires additional chemicals and generates secondary waste streams [6,7]. Consequently, considerable attention has been directed toward adsorption-based technologies, which combine high removal efficiency with operational simplicity, low energy requirements, and the possibility of utilizing inexpensive waste-derived sorbents. However, due to their low profitability and insufficient effectiveness at lower metal-ion concentrations, more cost-effective ways of managing them are being prospected [8]. A reasonable solution to these problems could be the use of biosorption, which is a far more resourceful and efficient technique for removing inorganic contaminants [5,9]. It is a simple and reversible technique, based on binding reactions of heavy metal ions with functional biomass groups. Many studies have shown its considerable potential for removing water and wastewater pollutants through sorption on plant materials, producing far fewer unwanted by-products in comparison with other chemical or physical remediation techniques [4,9,10,11,12].

**Figure 1:**
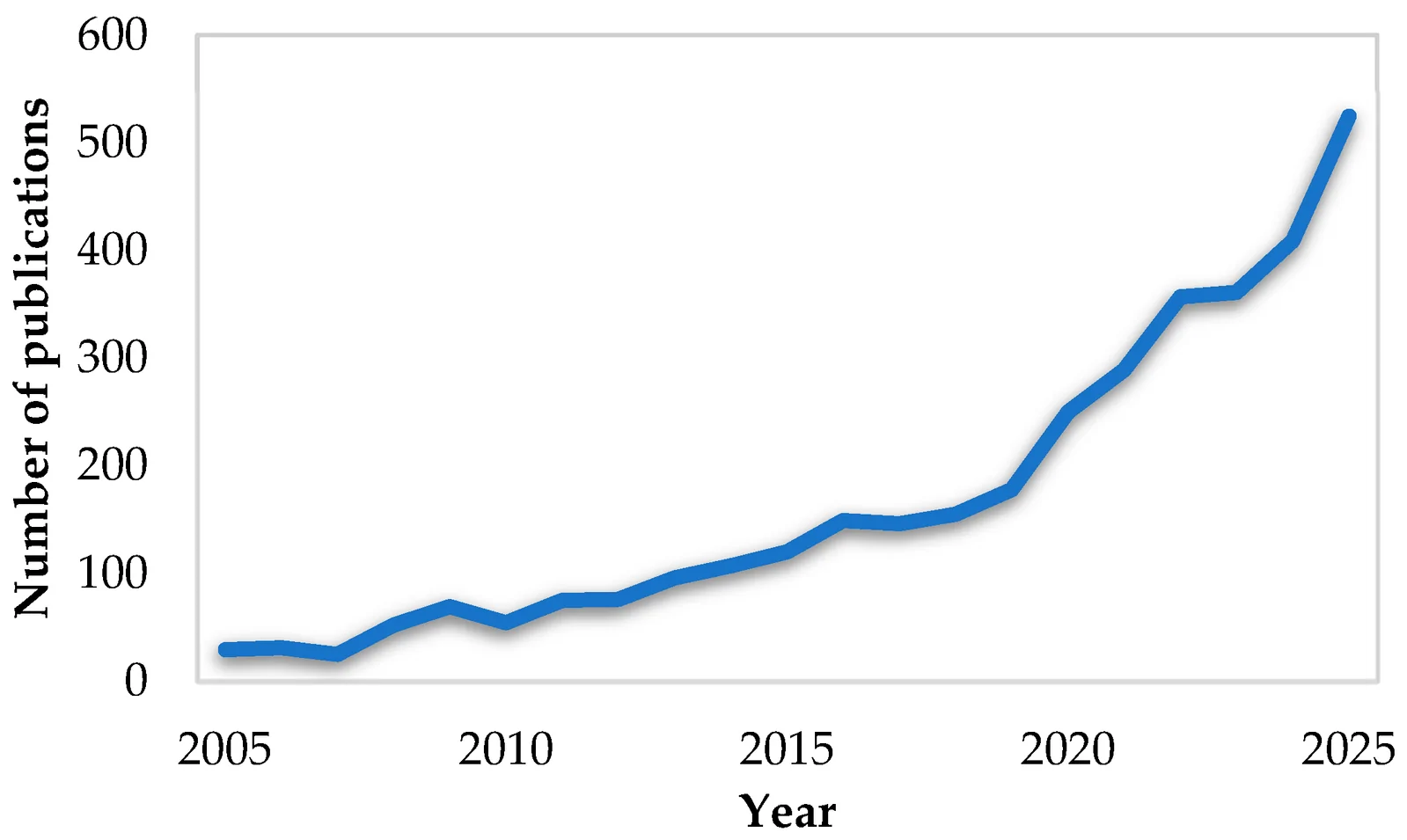
Annual number of publications on agricultural waste-derived adsorbents for water and wastewater treatment from 2005 to 2025 based on the Scopus database search performed on 7 October 2026. Document advanced search query: TITLE-ABS-KEY ((“agricultural waste” OR “agricultural residue” OR agrowaste OR “agro-waste”) AND (biosorbent OR adsorbent) AND (“water treatment” OR wastewater)). A total of approximately 3500 records were identified. The figure represents the authors’ own elaboration based on Scopus search results.

In 2024, China was the main global producer of fruit, with an average share of the fruit harvest of approximately 29%. It dominated global production of watermelons, apples and tangerines [13]. Fruit peels, pomace and other agri-food wastes have versatile properties, including high potential for use in removing heavy metal ions from aqueous solutions and wastewater. Some of the fruit and vegetables are processed into juices and jams, generating large amounts of waste in the form of pomace, which is used as animal feed or fertilizer, or burned. Fruit processing generates substantial quantities of pomace, typically accounting for approximately 25–35% of the processed fruit mass. Apple pomace is among the most abundant fruit-processing by-products worldwide and is generated in large quantities during juice and cider production [14]. The waste generation trend has been upward in recent years. Such a large amount of unmanaged waste poses an epidemiological threat due to the possibility of harmful microorganisms growing in the waste storage area. Moreover, factors such as light, heat or the presence of other oxidizing agents can lead to the degradation of many components, which are important for sorption processes. The sorption potential of plant waste is primarily due to its high content of cellulose, generally found in large quantities in agri-food industry wastes. Although fruit-processing residues constitute one of the most extensively investigated groups of biosorbent precursors, numerous other agri-food residues are generated worldwide in quantities that make them attractive feedstocks for large-scale water-treatment applications [15,16]. Agricultural by-products such as wheat straw, barley straw, oat hulls, rice husks, corn residues, sunflower husks, flax straw, and residues from pulse crops are rich in cellulose, hemicellulose, lignin, and various oxygen-containing functional groups capable of binding metal ions [15]. These materials are often underutilized and may be disposed of through open-field burning, land application, or low-value animal bedding uses [16,17]. In many regions, substantial quantities of lignocellulosic residues remain available after harvesting or food processing [16]. For example, flax cultivation generates significant volumes of straw that are not always fully utilized, while the processing of chickpeas, lentils, and other legumes produces hulls and plant residues rich in polysaccharides and surface functional groups [18]. Similar opportunities exist for cereal-processing by-products, including wheat and barley residues as well as oat hulls [17,19]. Due to their abundance, low cost, renewability, and high content of reactive hydroxyl, carboxyl, and phenolic groups, these materials have attracted increasing interest as biosorbents for the removal of heavy metals from contaminated waters [15,16,19]. Furthermore, proteins, lignin and hemicelluloses are important components involved in metal-ion complexation, which provides a basis for metal-ion removal [8]. The valorization of such agricultural residues through adsorption technologies simultaneously addresses two environmental challenges: the management of large biomass waste streams and the treatment of polluted water [15,16]. Consequently, agri-food residues represent an important component of the circular economy, enabling waste-to-resource strategies while reducing dependence on conventional commercial adsorbents [7,15,20]. A growing amount of research [11,12,21] demonstrates that fruit pomace, cereal residues, oilseed by-products, legume-processing wastes, and other lignocellulosic agri-food residues can serve as efficient, low-cost, and environmentally sustainable biosorbents for the removal of inorganic pollutants from water. In addition to their chemical characteristics, agri-food residues differ significantly in terms of their availability and market potential, which strongly influences their suitability for practical water-treatment applications. High-volume residues such as apple pomace, rice husks, sugarcane bagasse, sunflower husks and nut shells are generated annually in millions of tons [22,23,24] as unavoidable by-products of fruit processing, cereal milling and sugar production, ensuring a stable and low-cost supply chain for sorbent production. Their large-scale availability has already enabled several implementations: rice-husk-derived sorbents have been tested in fixed-bed columns for metal removal in rural water-treatment units [25]; sugarcane-bagasse-derived adsorbents and composites have been widely investigated for the removal of dyes and other contaminants from wastewater, demonstrating their potential as low-cost treatment materials [26]; and fruit-pomace-derived adsorbents have attracted growing interest for water-treatment applications, as recent studies have highlighted their potential for process integration and future scale-up for use in practical filtration systems [27]. These examples suggest that widely available agricultural residues have considerable potential for translation from laboratory-scale studies toward practical water-treatment applications, highlighting promising prospects for future scale-up and implementation. A clear increase in the annual number of publications can be observed, indicating growing scientific and technological interest in the valorization of agricultural residues as adsorbents for water and wastewater treatment (Figure 1).

A significant advantage of biosorption over other water-treatment methods is the versatility of modifying sorption materials, which greatly assists in process optimization and design. Since raw agri-food industry wastes often exhibit limited sorption capacity, researchers are investigating the possibility of modifying these materials [28]. The aim is to change the physicochemical properties, porosity, surface groupings or thermal stability of the biosorbent [29]. Different approaches to chemical, thermal and biological modifications are directed not only at improving the sorption capacity but also at the regeneration of the biosorbent [28]. Measures to increase the productivity of the fruit and vegetable industry are aimed at reducing the resources required for the production of these raw materials. Such investments in research and development can contribute to the implementation of new technologies for the processing and storage of products. In addition, promoting the use and marketing of by-products from the agri-food industry is proving to be an important issue [30].

The originality and contribution of this study are as follows: This study presents a targeted, mechanism-based synthesis that demonstrates how specific surface chemistries and modification methods (such as hydroxyapatite impregnation, esterification, and oxidative pretreatments) affect performance metrics derived from isotherm and kinetic analyses in both single- and multi-ion systems. It explicitly accounts for pH, dosage, and competitive ions. Recent reviews have extensively discussed agricultural wastes as adsorbents for a broad range of contaminants, including pesticides, pharmaceuticals, dyes, and other emerging pollutants. Other reviews have focused on specific feedstock groups, such as fruit peels or biochar-derived materials. In contrast, this review specifically addresses the removal of heavy metals using agri-food residues and their modified forms. Beyond summarizing adsorption capacities, this study also critically evaluates characterization methods, adsorption mechanisms, modification strategies, competing-ion effects, regeneration challenges, and scale-up limitations.

Additionally, it develops a decision framework that prioritizes high-volume agri-food residues, such as apple pomace, for scalable biosorbent production. This framework integrates performance standards with process-related constraints, including the translation of batch results to column scale, thereby addressing key implementation challenges highlighted in previous research. This approach aims to bridge the gap between laboratory-scale adsorption studies and practical water-treatment applications.

## 2. Literature Search and Selection

To write this article, a transparent literature selection strategy was applied. Relevant publications were identified using the Web of Science Core Collection, Scopus, and ScienceDirect databases. The literature search was conducted between January 2025 and February 2026 and covered publications from 2000 to 2026. The following search terms and combinations were used: “agri-food waste” OR “agricultural waste” OR “fruit pomace” OR “food processing residues”; AND “adsorption” OR “biosorption”; AND “heavy metals” OR “metal ions” OR “inorganic pollutants”; AND “water treatment” OR “wastewater treatment”; AND “biochar”, “surface modification”, or “chemical modification”. Studies were included if they: (i) investigated agri-food residues or biomass-derived materials as adsorbents for inorganic pollutants; (ii) reported adsorption performance data, such as adsorption capacity, removal efficiency, kinetic parameters, or isotherm analyses; and (iii) described the physicochemical properties or modification methods of the biosorbents. Studies focusing exclusively on organic pollutants, dyes, pharmaceuticals, or non-aqueous systems were excluded unless they provided mechanistic information directly relevant to metal-ion adsorption. This article should be regarded as a narrative review supported by a structured literature search. Following the database searches, duplicate records were removed before further evaluation. Particular emphasis was placed on studies reporting adsorption performance, adsorption mechanisms, physicochemical characterization, and modification strategies of agri-food-derived adsorbents. Approximately 550 publications were initially identified, of which approximately 150 were retained for detailed analysis after applying the eligibility criteria. Materials outside the primary agri-food scope, such as lignite, forestry-derived biomass, or municipal-solid-waste-derived biochars, were included only when used to illustrate analytical methodologies, adsorption models, or modification approaches relevant to biomass-based adsorbents. These materials were not considered representative agri-food biosorbents and were not included in the further evaluation.

## 3. Methods for Studying the Sorption Activity of Biomass

### 3.1. Material Characterization

#### 3.1.1. Determination of the Biosorbent Main Composition

The first step in estimating the overall composition of a material is to determine its moisture content. For example, the use of fruit or vegetable pomace requires consideration of the fact that it has a fairly high moisture content, which makes it prone to fermentation, reducing its value and eliminating the possibility of further use due to spoilage [31]. For this reason, it must always be dried before use. Subsequently, such raw material must be stored in suitable conditions that prevent the entry of moisture from the environment. Moisture content depends on the type of material and processing technology. According to the literature, the moisture content of dried apple and beet pomace ranged from 3.9 to 10.8% and 10.1%, respectively [32], while coffee husk had a moisture content of more than 17% [33]. It is also crucial to determine the presence of major components involved in adsorption processes. In order to identify economically and environmentally friendly materials that could serve as biosorbents, much work has focused on agricultural waste, such as fruit/vegetable pomace, nut shells and forest residues [4]. These raw materials have extremely rich compositions, including cellulose, hemicellulose and lignin. These natural polymers are associated with various functional groups, which are involved with interactions with pollutant molecules, such as metal ions. These reactions involve complex mechanisms, including complexation, ion exchange, precipitation and physical adsorption. The functional groups important in this case include hydroxyl, carbonyl, carboxyl, aldehyde, ether and phenolic groups [34]. Determination of their qualitative and quantitative composition can be performed by Fourier-transform infrared (FT-IR) spectroscopy, ultraviolet-visible (UV-VIS) spectroscopy, elemental analysis, chromatographic and many other techniques.

#### 3.1.2. FT-IR Spectroscopy

FT-IR spectroscopy is used to identify individual functional groups present on the surface of biosorbents and to determine whether the molecules of the contaminant bind to these groups, making it possible to prove which of them are responsible for a material’s sorption activity [11,35,36]. Kayranli, in his study on mechanisms of cadmium ion removal by lignocellulosic pistachio, peanut and almond residuals [37], registered FT-IR spectra of these materials to approximate their sorption mechanism. As with all plant materials, their spectra were very complex. Broad peaks at wavenumbers in the range 3272–3331 cm^−1^ were assigned to symmetric stretching vibrations of hydroxyl groups originating from cellulose, hemicellulose and lignin. Further, slightly less intense signals were identified at wavenumbers of 2843, 2918 and 2893 cm^−1^, arising from stretching vibrations of –OH and C–H groups found in carboxylic acids, aldehydes or alkanes. Peaks occurring at wavenumbers of 1722 and 1728 cm^−1^ were assigned to vibrations arising from C=O bonds of carboxylic groups. Signals at 1597 or 1606 cm^−1^ wavenumbers arose from C=O and C–O stretching vibrations of carboxyl and carbonyl groups. In the fingerprint region (wavenumbers in the range of 1500 to 500 cm^−1^), a large number of signals were found on the spectra of all three materials. Bands at 1416–1251 cm^−1^ wavenumbers were attributed to stretching vibrations of –CH_3_ and –CH_2_ groups in alkanes, alcohols and carboxylic acids and their C=C aromatic groups, all of which are present in cellulose, lignin or hemicellulose. Peaks at 1026, 1018 and 1011 cm^−1^ were assigned to O–H and C=O stretching vibrations present in cellulose molecules . Of all the bands present in the described spectra, the peaks at the wavenumber ranges of 1011–1026 cm^−1^ were the most intense, along with those at 3272–3331 cm^−1^ and 1722–1728 cm^−1^. This indicated that the surface of the studied sorbents contained the highest amounts of hydroxyl, carboxyl and carbonyl groupings, which is crucial when applying these materials for the removal of metal ions from solutions [37]. Chand and colleagues used FTIR to determine the main functional groups present on the surface of apple pomace and their differences after sorption of cadmium(II) ions [38]. According to a theory that bands of functional groups involved in adsorption disappear or decrease in intensity after sorption, it was concluded that –OH and –NH groups in bonded form, the –C=O group present in esters, the –CO carbonyl group, and –C–C– and amine group were responsible for binding metal ions [38]. FTIR spectra may indicate the participation of functional groups in metal binding but do not by themselves prove the dominant removal pathway.

#### 3.1.3. Imaging with Scanning Electron Microscopy

Another important technique for studying the adsorption potential of materials is scanning electron microscopy (SEM). It makes it possible to visualize the morphology of an adsorbent and to determine, among others, the shape and size of protrusions and open pores. This is possible thanks to the good resolution and excellent depth of focus that can be achieved by using SEM. The topography contrast could also reveal the presence of so-called pockets, increasing the specific surface area of the absorbent and indicating an ability to attach metal ions and other impurities compared to smooth, homogeneous substrates [39]. Sheikh and others investigated the potential use of *Allium cepa* seeds for the adsorption of metal ions. The first step in their research was to characterize the surface morphology of this biosorbent by SEM. The most important observation was the presence of a large number of macropores and micropores throughout the sorbent surface. This feature is crucial in assessing the sorption potential of biomass, as it increases the surface area available for sorption [40]. Although SEM analysis provides valuable information on the surface morphology of the adsorbent, morphological observations alone cannot unequivocally identify the adsorption mechanism. Therefore, SEM results should be interpreted together with adsorption performance, kinetic modeling, and other physicochemical characterization techniques [41]. SEM observations mainly provide information on the surface morphology and cannot independently identify adsorption mechanisms.

#### 3.1.4. Brunauer–Emmet–Teller (BET) Surface Area Analysis

A large surface area is considered one of the most important characteristics of a good adsorbent; therefore, determining its size is often the first step in studying a material with potential sorption properties. A higher surface area is generally associated with a larger number of active functional groups available for interaction with metal ions [42]. BET (Brunauer–Emmett–Teller) analysis, based on the determination of adsorption isotherms for gases (e.g., carbon dioxide), is often used for this purpose [43]. Cheng et al. studied the sorption properties of bio-modified lignite, in comparison with unmodified lignite. The results of their analysis indicated that the BET specific surface area increased after modification, from 1.81 to 5.66 m^2^·g^−1^, also increasing its sorption capacity for Cu(II), Mn(II), Cd(II) and Hg(II) ions severalfold [42]. BET surface area analysis results for low-cost wood biosorbents (banana pith, bamboo and pineapple stem) in a study by Rajesh and Jeeru were about 3.5, 5.7 and 9.1 m^2^·g^−1^, respectively [44]. Chemical treatment of these materials with H_3_PO_4_ and NaOH allowed these values to be increased significantly, to about 100.2, 116 and 116 m^2^·g^−1^, respectively.

Although the BET surface area is one of the most frequently reported characteristics of adsorbents, it does not always represent the surface area that is accessible under aqueous adsorption conditions. Many biomass-derived sorbents may undergo hydration and partial swelling during contact with water, which can influence pore accessibility, diffusion pathways, and the availability of surface functional groups involved in adsorption [45]. Consequently, adsorption measurements based on probe molecules dissolved in aqueous media may provide complementary information regarding the effective adsorption surface available during practical operation. Therefore, surface-area values obtained by BET analysis should be interpreted together with adsorption-performance data and swelling behavior.

#### 3.1.5. Thermogravimetric Analysis

The most popular thermo-analytical methods are thermogravimetric analysis (TGA), differential thermal analysis (DTA) and differential scanning calorimetry (DSC). The purpose of TGA is to record changes in the mass of a substance as a function of temperature [46]. The DTA technique is used to determine temperature differences between the test sample and a reference substance. If the temperature of the test sample is higher than that of the reference sample, we are dealing with an exothermic reaction and a peak with an upward-directed maximum is observed. In the DSC technique, the proportionality of the recorded signal to the difference in heat fluxes makes it possible to directly measure the heat capacity of the sample, and to qualitatively/quantitatively observe the course of material transformations [47]. The amount of heat that needs to be supplied to equalize the temperature between the sample and the environment is recorded as a heat effect in the form of a peak, where its area equals an enthalpy value of this transformation [46], which is used to identify individual substances. For plant-derived materials, such as fruit, herb or vegetable waste, TGA is particularly useful for determining their stability. In their unprocessed form, their storage time is relatively short due to their high water content, while overly harsh heat treatment can cause valuable chemical compounds to decompose [48]. Thermogravimetric analysis provides information not only on thermal stability but also on the composition and thermal behavior of lignocellulosic biomass. In an inert gas atmosphere, hemicelluloses are the first to decompose at 250–300 °C, followed by celluloses between 300 and 350 °C, and lignin between 300 and 500 °C [47]. The information obtained strongly depends on the applied heating rate. Increasing the heating rate generally shifts TG and DTG peaks toward higher temperatures because the sample experiences a thermal lag and requires a higher temperature to achieve the same degree of conversion. As a consequence, the decomposition stages may partially overlap, affecting the apparent temperatures of hemicellulose, cellulose, and lignin degradation [49]. Therefore, the heating rate should always be reported when comparing thermogravimetric data obtained from different studies.

**Figure 2:**
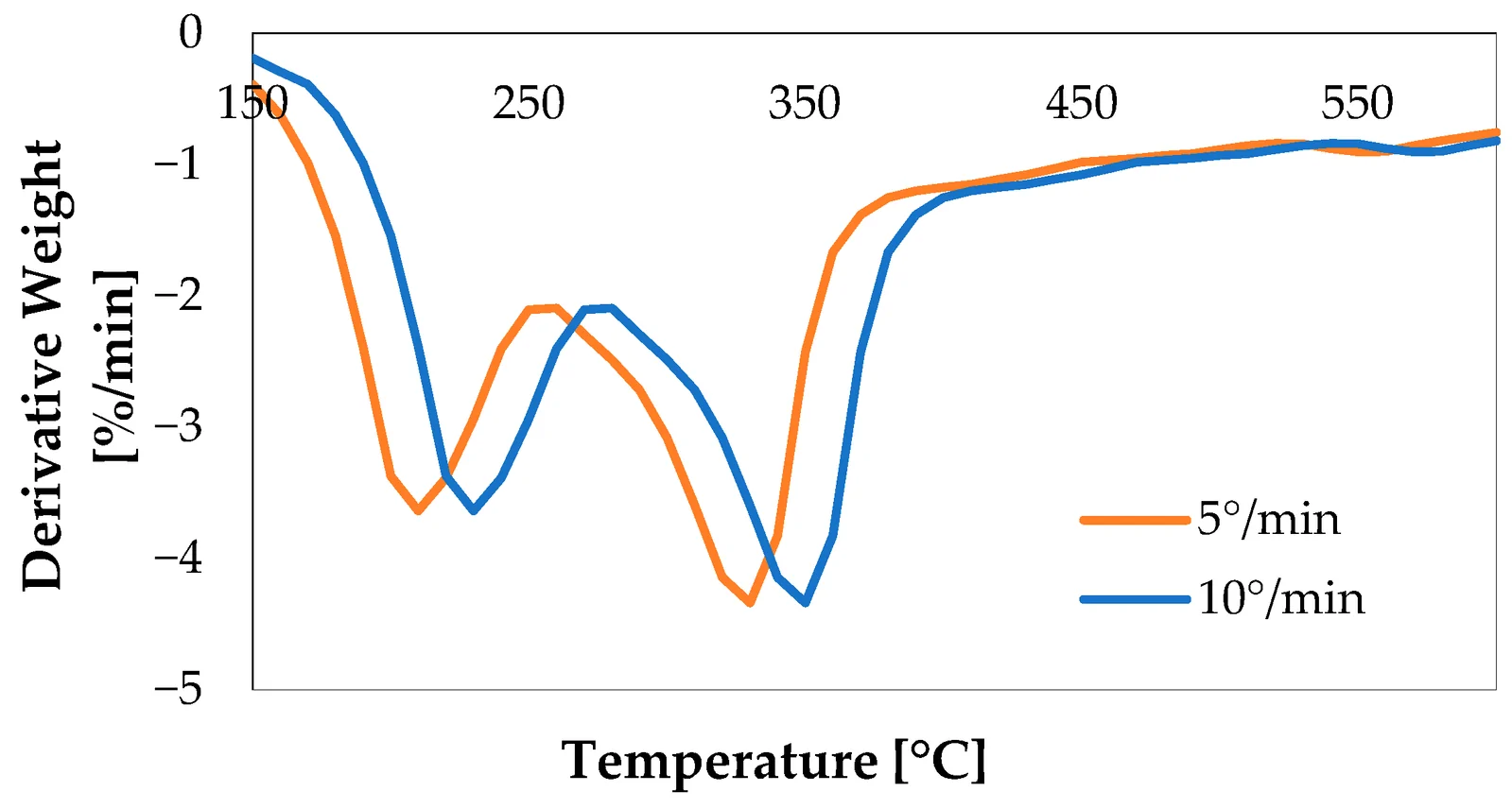
Schematic DTG curves of lignocellulosic biomass at different heating rates (schematic illustration based on [49]).

Emiola-Sadiq et al. investigated agricultural and forestry biomass residues at different heating rates and demonstrated that increasing the heating rate significantly affected the devolatilization behavior and kinetic parameters calculated from TGA measurements. The authors observed that characteristic decomposition peaks shifted to higher temperatures as the heating rate increased, highlighting the importance of experimental conditions during thermal characterization (Figure 2) [49].

Derivative thermogravimetric (DTG) curves are particularly useful because they facilitate the identification of individual biomass fractions. Hemicellulose typically decomposes first and produces a DTG peak around 200–320 °C; cellulose exhibits a sharper and more intense peak, commonly observed between 300 and 400 °C; and lignin decomposes over a considerably broader temperature range extending approximately from 200 °C to over 700 °C [49]. The relative areas and intensities of these DTG peaks can be used to estimate the proportion of the major lignocellulosic components present in biomass.

V. Nejadshafiee et al. conducted a TG/DTG analysis of sultone-modified magnetic activated carbon and compared its TG results with those of activated carbon without modification. On the DTA curve of the modified sorbent, the first mass loss, accompanied by an endothermic peak, occurred at temperatures up to 110 °C, caused by water evaporation. Next, two exothermic signals at 300 and 600 °C, with mass losses of 4 and 39%, respectively, were likely due to decomposition of organic matter. On the other hand, it was noted in the TG curves that, compared to activated carbon, which reached a few percent of its initial mass after exceeding temperatures of about 750 °C, modified adsorbent retained about 55% of its initial mass in the temperature range of 600–1000 °C [36].

Boundzanga et al. [50] obtained TG/DSC plots for different plant materials in an argon atmosphere, ranging from room temperature to 800 °C. Each of the materials was characterized by water loss in the temperature range of 80–120 °C, resulting in a loss of about 10% of each material’s weight. Overall, at 800 °C, the weight loss oscillated around 75% for all samples. The DSC curve of olive pomace consisted of two very distinct peaks at temperatures of 280 °C and 340 °C, from hemicellulose and cellulose, respectively. Miscanthus, in a DSC curve, showed peaks corresponding to hemicellulose and cellulose at 300 °C and 358 °C, respectively, tomato plant peaks at 250 °C and 315 °C, and poplar wood peaks at 290 °C and 350 °C [50].

#### 3.1.6. Influence of Adsorbent Physical Form on Adsorption Performance

In addition to chemical composition and surface chemistry, the physical form of the biosorbent can significantly influence adsorption performance. Numerous studies report adsorption capacities using finely ground powders because they provide short diffusion paths and a large exposed surface area [51]. However, powdered adsorbents often present challenges during practical implementation, including difficult separation from treated water, particle loss, limited mechanical strength, and high pressure drops in fixed-bed systems. Pelletized, granulated, immobilized, or composite biosorbents may exhibit lower apparent adsorption capacities on a mass basis, but they frequently provide superior operational performance [52]. Improved hydraulic properties, mechanical stability, easier regeneration, and reduced clogging making them more suitable for continuous fixed-bed applications [53]. Consequently, adsorption capacity alone should not be considered sufficient for adsorbent selection.

Additionally, composite porous materials prepared by combining agri-food residues with inorganic components such as calcium-rich materials, eggshell-derived phases, biochar minerals, hydroxyapatite, bentonite, kaolinite, or layered double hydroxides have attracted increasing interest [15,20]. The incorporation of these mineral phases may improve pore structure, increase surface heterogeneity, enhance mechanical durability, and create new adsorption mechanisms, including ion exchange, surface complexation, and electrostatic interactions. As a result, composite biosorbents often demonstrate improved adsorption capacity and operational stability compared with their unmodified counterparts, making them more suitable for practical water-treatment applications [16,19].

### 3.2. Sorption Performance

In the literature on the sorption activity of sorbents against different contaminants, two main techniques are encountered: batch and fixed-bed column sorption [54]. The former occurs in scientific papers more than twice as often (according to the Web of Science database), which is likely because less sorbent material and less operational time are required [55,56]. On a laboratory scale, it is simply a more cost-effective method by which many important parameters concerning sorption and sorbent–sorbate interactions can be developed. Subsequently, the information obtained can be adopted on a larger, industrial scale, where the column technique is more commonly used [54]. 

**Figure 3:**
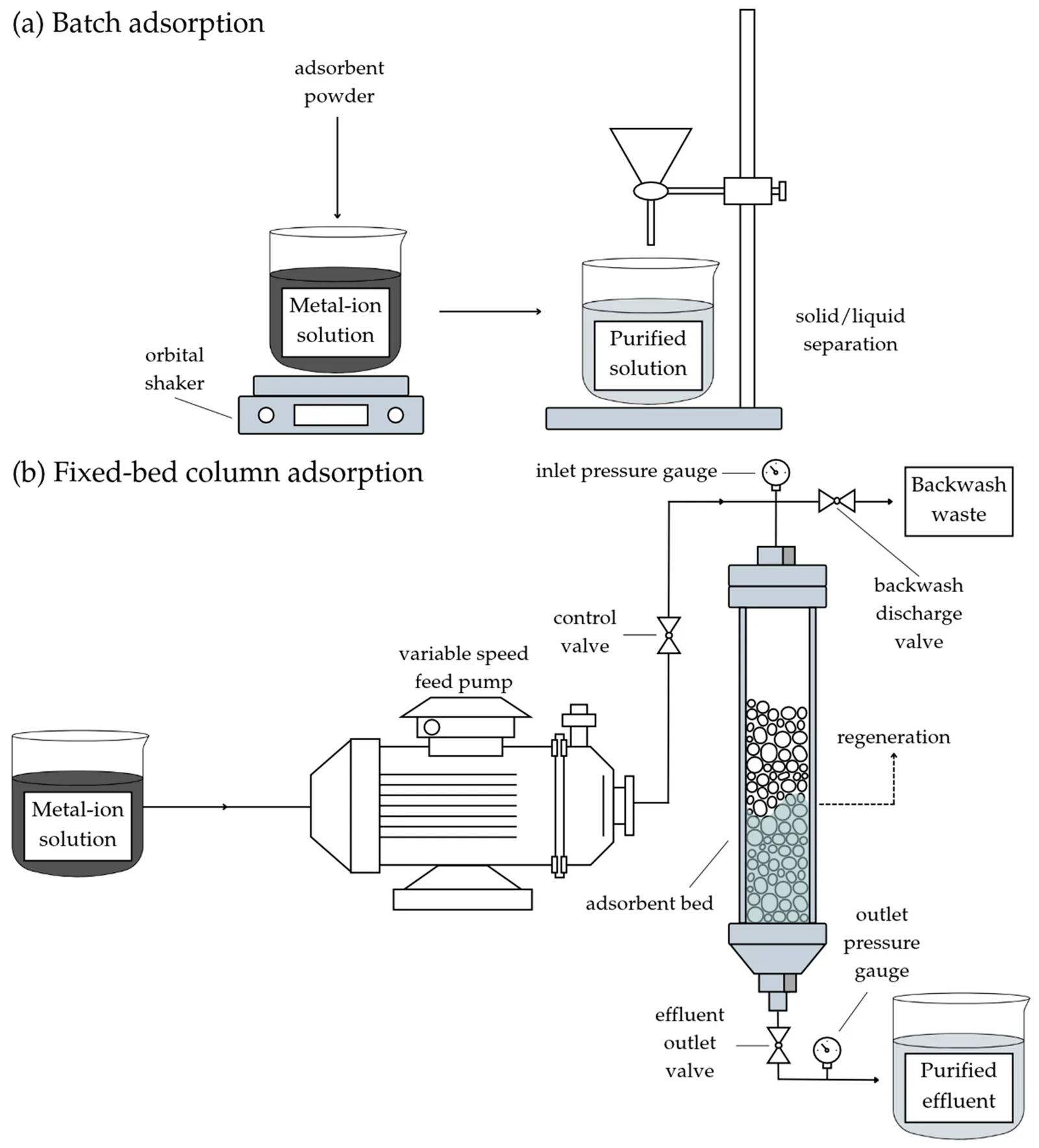
Schematic of conducting a sorption experiment using the batch sorption method and the fixed-bed column method (own work based on [54]).

Batch sorption experiment methods are used in order to obtain the adsorption isotherms or kinetic equilibrium constants of metal ions on an adsorbent’s surface. It is necessary to understand these parameters to determine the mechanisms that control sorption processes on any material. Figure 3a shows a schematic of a sorption test using the batch technique, known as the static sorption method. Adsorbate solution and adsorbent dose are mixed in a glass flask/bottle on a horizontal shaker at a specific speed, for a sufficient contact time.

There are several widely studied crucial parameters that have an impact on batch adsorption performance. Contact time is often considered the most important, which depends on both the type of contaminant and adsorbent. It has an equilibrium value specific to a particular system, beyond which any further increase does not increase the rate of adsorption [54]. Until it is reached, the degree of adsorption mostly increases with contact time. The results of Ghasemi et al., Wahynuhadi et al. and Chen et al. have proven such a relationship, with contact times of 50, 75 and 50 min, respectively; increasing it beyond these values did not increase the sorption efficiencies [42,57]. Another parameter is the adsorbent dose. As the mass of an adsorbent increases, so does the efficiency of metal-ion removal. This is due to an increase in the sorption area and, thus, the number of binding sites available to metal ions [58]. The solution pH is another important parameter influencing adsorption performance, which affects the adsorption efficiency. Low pH results in lower sorption efficiency due to a high concentration of hydrogen ions, which are in competition with adsorbate ions for adsorption sites of functional groups present on the sorbent surface [59]. At least one more important parameter, the initial adsorbate concentration, should also be taken into account when designing an efficient adsorbent. In general, higher concentrations of an adsorbate in aqueous solution reduce the adsorption efficiency of an adsorbent [40,60]. In addition to affecting the surface charge of the adsorbent and the ionization of functional groups, pH strongly influences metal speciation in aqueous solutions. Many metal ions occur as different dissolved species depending on pH, including free cations, hydrolyzed complexes, and precipitated hydroxides. Consequently, changes in adsorption performance with pH may result not only from changes in sorbent surface chemistry but also from changes in metal speciation and solubility. For example, metals such as Cu(II), Pb(II), Cd(II), and Cr(III) may form hydrolyzed species at elevated pH values, whereas excessive pH increases may promote hydroxide precipitation. Therefore, adsorption data should always be interpreted together with metal speciation diagrams and precipitation equilibria.

The fixed-bed column (continuous) adsorption method is commonly used to treat mixtures of industrial wastewater [61]. This technique involves passing a solution of a given contaminant through a column packed with a sorbent (which is a fixed, solid bed), after which a treated medium (water/wastewater) is discharged (Figure 3b) [54]. The most important parameters required for the design of a fixed-bed adsorption system are the dynamics and adsorbent lifetime data. Operating parameters such as mass-transfer resistance and the rate of the mixture passing through the column must be considered [62]. On a large scale, wastewater treatment plants require a continuous mass flow due to a high volume of wastewater, hence the significant advantage of this sorption method over the batch technique. In order to study a mechanism of the sorption process on a particular fixed bed, scientists use mathematical equations to calculate the amount of contaminant at which the sorbent will require regeneration [54]. To remove copper(II) ions using the column adsorption method, the literature reported the use of coconut shells [63], bentonite with immobilized chitosan [55] and activated carbon from waste rice husks [64], among others. Examples of adsorbents for nickel(II) ion removal include activated carbon from sugarcane bagasse [65] and tea factory waste [66].

To describe adsorption behavior under continuous-flow conditions, several fixed-bed kinetic models are commonly used. The Thomas model is among the most frequently applied models. It is based on second-order reversible reaction kinetics and assumes Langmuir-type adsorption behavior, negligible axial dispersion, and the absence of significant external and internal diffusion limitations [67]. It is widely used to estimate column adsorption capacity and predict breakthrough behavior. The Yoon–Nelson model provides a simplified approach based on the probability of adsorbate breakthrough and allows for estimation of the time required for 50% saturation of the column. Moreover, the Adams–Bohart and Bed Depth Service Time (BDST) models are particularly useful during the early stages of fixed-bed design and scale-up. These models provide information that cannot be obtained from batch experiments alone and therefore constitute an important step in the development of practical adsorption systems [67].

Although batch adsorption experiments provide valuable information regarding adsorption capacity, equilibrium behavior, and adsorption mechanisms, their results frequently overestimate the performance achievable under continuous-flow operation. In batch systems, adsorbent particles remain in constant contact with the solution under ideal mixing conditions, whereas fixed-bed columns are affected by mass-transfer limitations, axial dispersion, channeling, hydraulic residence time, and non-uniform flow distribution. Consequently, materials exhibiting excellent adsorption capacities in laboratory batch experiments may demonstrate substantially lower usable capacities under dynamic column conditions. Therefore, the adsorption capacity obtained from batch studies should not be directly interpreted as an indicator of industrial performance without validation through breakthrough-column experiments. Fixed-bed breakthrough studies should be regarded as an essential step in evaluating practical applicability. Table 1 provides an overview of recommended indicators that are essential to evaluate the potential of process scale-up.

#### Quantification Methods of Adsorbed Substances

Researchers highlight atomic absorption spectroscopy (AAS), inductively coupled plasma mass spectrometry (ICP-MS) and ultraviolet-visible (UV-Vis) spectroscopy among the techniques used to quantify metal-ion content in purified solutions.

AAS is based on Beer’s law, which states that the absorbance value of a solution is directly proportional to the concentration of a substance [68]. It is one of the most widely used absorption techniques in environmental analysis, mainly because of its easy operation and low operating costs [69]. Examples of ICP-MS spectra of solutions containing a mixture of Sr(II), Y(III) and Zr(IV) cations before and after sorption with cation exchange were presented by Vasylyeva et al. [70]. There are many advantages in favor of using ICP-MS, which include low interference, high precision and very low detection limits for substances. Integration with separation technologies that allow for the separation and analysis of ions in different valence states is also an important advantage. The disadvantages of this technique include the high operating and apparatus costs involved [71]. Another technique for the qualitative and quantitative detection of individual metal ions, which requires an apparatus that is slightly less specific than previous techniques, is UV-Vis spectroscopy. In general, this technique involves the production of metal-ion complexes that are detected by UV-Vis spectroscopic methods. An example of such an assay is the use of PAR (4-(2-pyridylazo)resorcinol) salt to complex Cu(II), Zn(II), Ni(II) and many other ions. The result is the immediate production of an orange-red coloration of the solution, whose absorbance values are read with a spectrometer at the analytical wavelength [72].

### 3.3. Modeling

#### 3.3.1. Determination of Kinetic Parameters of the Sorption Process

**Figure 4:**
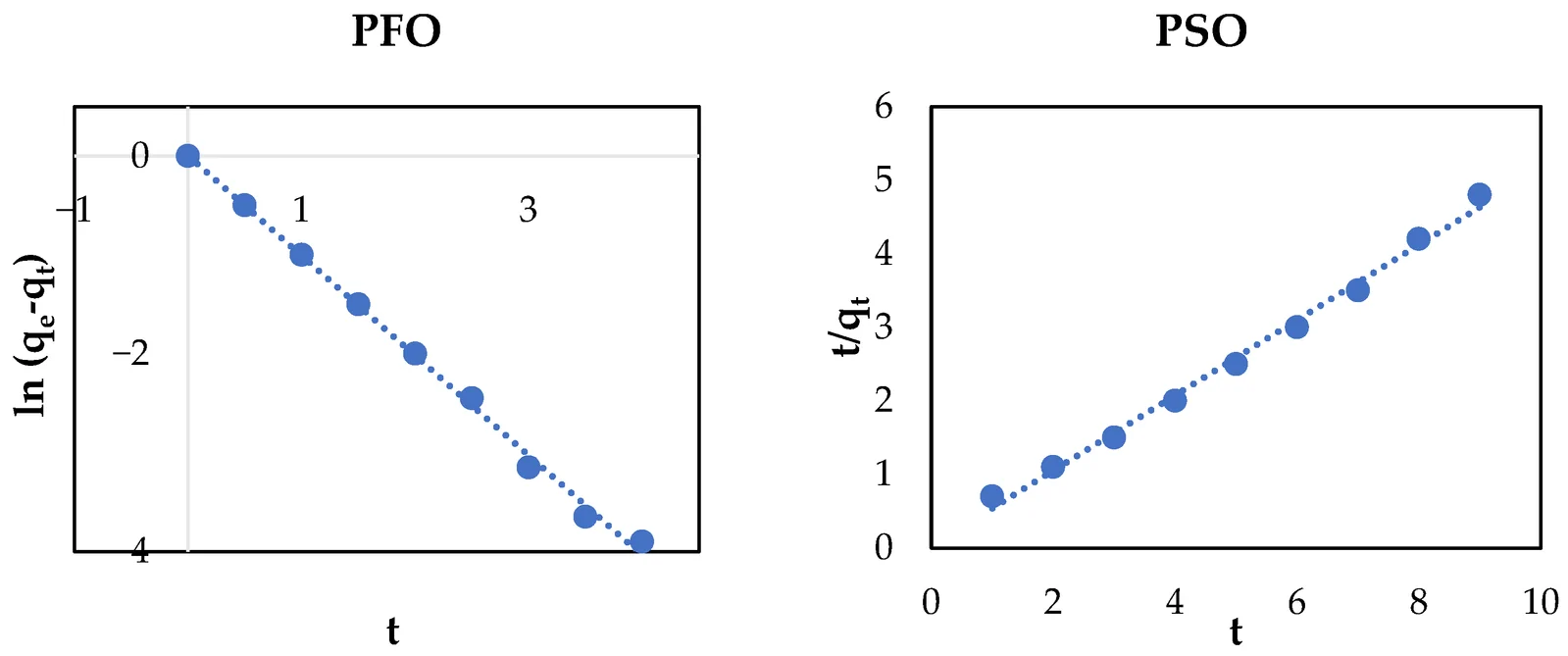
Typical adsorption kinetics (PFO—pseudo-first-order, PSO—pseudo-second-order) (schematic illustration based on [77]).

Evaluation of sorption performance under practical conditions and rational adsorbent design require a thorough understanding of the kinetic parameters of the adsorption process using a particular sorption material. Correctly selected kinetic models and measurement accuracy provide valuable information on the sorption mechanism. The most commonly used kinetic models are pseudo-first-order and pseudo-second-order models (Figure 4). The former was developed by Lagergren, and it assumes a directly proportional dependence of the reaction rate on the difference between the equilibrium concentration of an adsorbate in the solid phase of the adsorbent and its instantaneous concentration in this phase [73]. In the second model, developed by Ho, the reaction rate is proportional to the square of the difference between the equilibrium concentrations in the adsorbate [74]. The accuracy of matching the equation of a model with the experimental results is confirmed by the determination of correlation coefficients and a correspondence of the experimental maximum sorption capacity value with the calculated one. Data from the experimental literature mostly report a better fit of the pseudo-second-order kinetic model for metal-ion adsorption onto plant-derived adsorbents. For example, Huang et al. obtained a correlation coefficient of 0.999 for the adsorption of Pb(II) and Cu(II) ions using the pseudo-second-order model, whereas the pseudo-first-order model yielded a value of 0.946. In addition, the equilibrium adsorption capacity calculated from the pseudo-second-order model was consistent with the experimental value [75]. Although such observations suggest that the pseudo-second-order model adequately describes the adsorption kinetics, they do not by themselves prove that chemisorption is the rate-controlling mechanism. Therefore, the model should be regarded primarily as an empirical kinetic description, while the actual adsorption mechanism should be supported by additional evidence, such as spectroscopic characterization, thermodynamic analysis, or mass-transfer investigations [76].

Experimental literature data mostly show a better fit of the pseudo-second-order kinetic equation for metal-ion adsorption on plant waste materials. For example, Huang et al., in a study of the adsorption kinetics of Pb(II) and Cu(II) ions, obtained a correlation coefficient of 0.999 after using this model, while the use of a pseudo-first-order model yielded a value of 0.946. Also, the calculated value of the maximum sorption capacity under equilibrium conditions was in agreement with a value resulting from an experiment [75].

Although pseudo-first-order and pseudo-second-order models remain the most frequently applied kinetic approaches, advanced kinetic interpretation often requires complementary models. The Elovich model is commonly employed to describe adsorption on energetically heterogeneous surfaces, whereas the Weber–Morris intraparticle diffusion model enables evaluation of mass-transfer limitations and the identification of diffusion-controlled adsorption stages. The Boyd model can further distinguish between film diffusion and intraparticle diffusion as the rate-limiting step [77].

#### 3.3.2. Analysis of Sorption Isotherms

**Figure 5:**
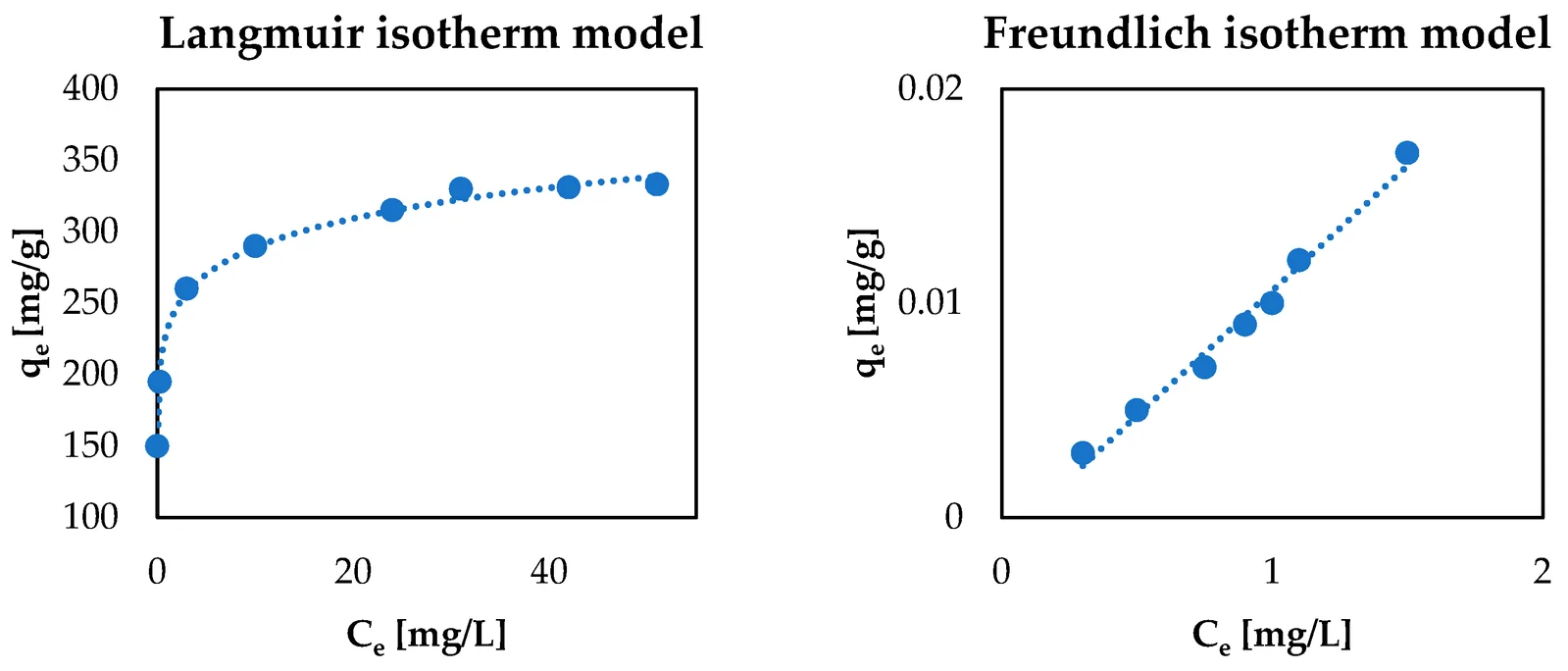
Conceptual representation of the Langmuir and Freundlich isotherms in q_e_ versus C_e_ coordinates. (schematic illustration based on [80,83]).

**Figure 6:**
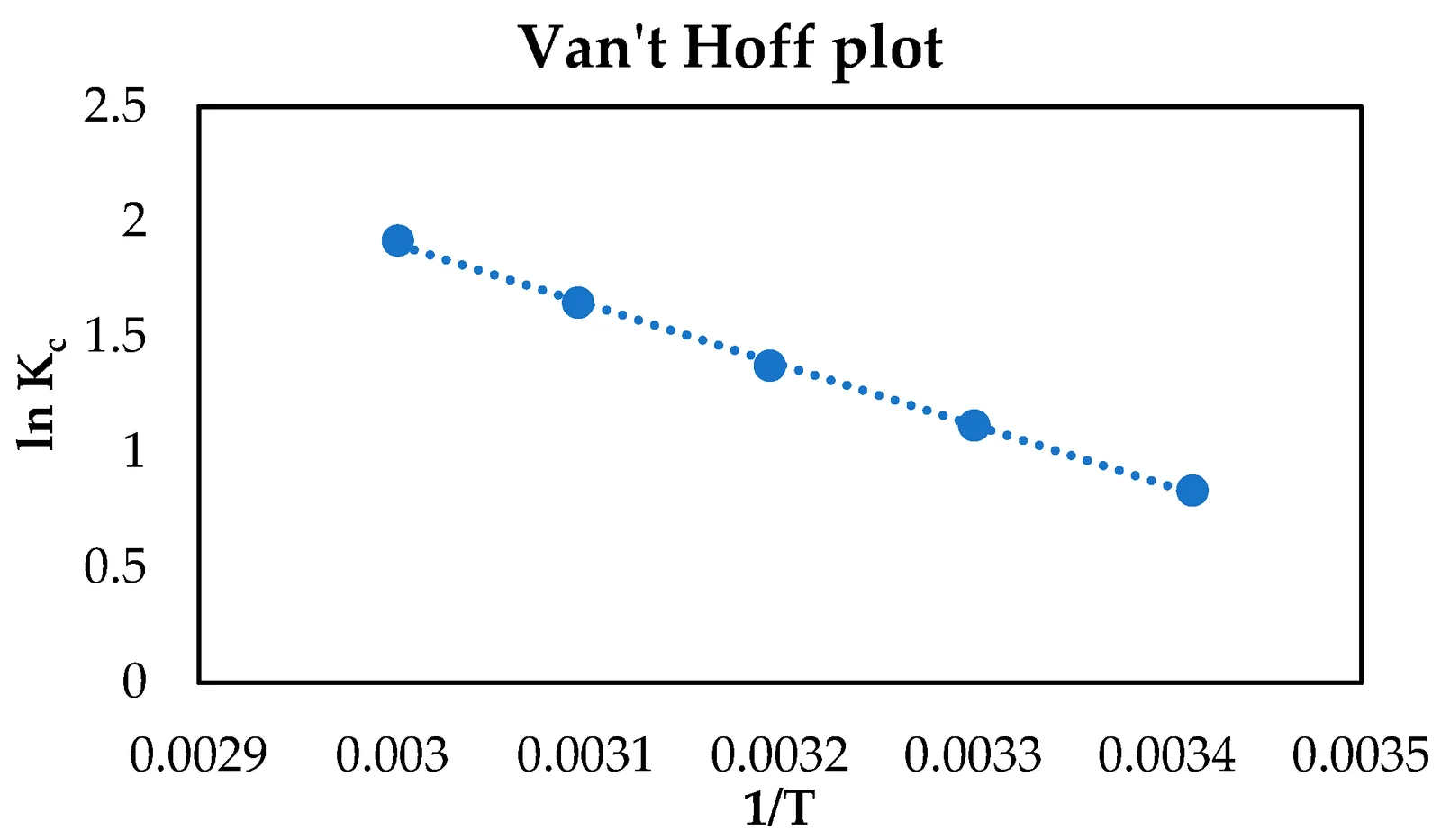
Representative van’t Hoff plot used to determine adsorption thermodynamic parameters (schematic illustration based on [86]).

One of the bases for determining the mechanism of the sorption process of a pollutant on a particular sorbent is an estimation of the adsorption isotherm model. There are many established adsorption models that describe the nature of this process, including the Langmuir and Freundlich isotherm equations. These models are fitted to experimental results, considering the assumed distribution of functional groups of the adsorbent involved in adsorption, the number of layers of contaminant molecules that can bind to the surface or the adsorption reversibility [78]. Beyond providing a mathematical description of equilibrium behavior, adsorption isotherms may also offer valuable insights into the underlying adsorption mechanisms. As for fitting an equation to the isotherms of sorption on sorbents of plant origin, the most frequently described research works suggest agreement with the equations of the Langmuir and Freundlich isotherms (Figure 5) [11,71,79]. The curves shown in Figure 6 are conceptual illustrations intended to demonstrate the characteristic shapes of the Langmuir and Freundlich isotherms and do not represent experimental data from a specific adsorption system. Monolayer adsorption is assumed by the first model. A good fit to the Langmuir model generally suggests that adsorption occurs predominantly on a finite number of energetically similar sites and may indicate the formation of a relatively uniform adsorption layer [80]. In biomass-derived sorbents, such behavior is often associated with specific interactions between metal ions and surface functional groups, including ion exchange and surface complexation. Its graphical representation on the basis of experimental sorption parameters makes it possible to determine key parameters describing the sorption isotherm: the maximum sorption capacity and a separation coefficient. While the first parameter makes it possible to estimate the amount of adsorbate that can be adsorbed on the studied material, the second characterizes the shape and nature of the isotherm, determining its favorable course, the reversibility of the process and the linearity of its equation [81]. Agreement with the Freundlich model is commonly interpreted as evidence of surface heterogeneity and the coexistence of adsorption sites with different binding energies [80]. Such behavior is frequently observed for lignocellulosic biosorbents due to the presence of multiple functional groups originating from cellulose, hemicellulose, lignin or proteins. This equation includes the Freundlich intensity parameter (n) or its reciprocal (1/n), which provides information about adsorption favorability and surface heterogeneity. In general, values of 1/n between 0 and 1 (or equivalently n > 1) indicate favorable adsorption and suggest that adsorption capacity increases with increasing adsorbate concentration. However, Freundlich parameters alone do not provide sufficient evidence to distinguish between physical adsorption and chemisorption. Such interpretations should be supported by thermodynamic analyses and/or adsorption energy calculations [80,82]. In order to correctly fit the experimental data to the adsorption isotherm models used, linear regression methods are used. Modern adsorption studies increasingly employ nonlinear regression because linearization may introduce statistical bias and alter the distribution of experimental errors. Therefore, model selection should not rely exclusively on correlation coefficients (R^2^ values). Additional statistical criteria, including residual analysis and error functions such as the root mean square error (RMSE), chi-square (χ^2^), or sum of squared errors (SSE), may provide a more reliable assessment of model performance [80,81].

In the case of sorption of copper(II) ions by biotransformed lignite, the Langmuir isotherm model proved a better fit compared to the Freundlich model, obtaining correlation coefficient values of 0.95 and 0.91, respectively, while Cd(II) removal efficiencies reached up to 96.6% for sunflower seed hulls [42]. On the other hand, according to the experimental data of Asimbaya et al., adsorption of zinc(II) ions by lignocellulosic biomass occurred according to the Freundlich isotherm equation, obtaining a correlation coefficient value above 0.96 [35]. The process of ion adsorption on plant materials is highly complex—no single isotherm model can definitively describe a certain group of adsorbents.

Besides the classical Langmuir and Freundlich models, adsorption equilibrium is frequently analyzed using several additional isotherm models that account for surface heterogeneity and non-ideal adsorption behavior. The Temkin model incorporates adsorbate–adsorbent interactions and assumes that the heat of adsorption decreases linearly with surface coverage. The Dubinin–Radushkevich model allows for the estimation of adsorption energy and is often used to evaluate whether adsorption may proceed through physical or chemical interactions. More flexible three-parameter models, including the Sips and Redlich–Peterson equations, combine features of both Langmuir-type and heterogeneous-surface adsorption and often provide improved fitting of experimental data over a wide concentration range [80,81]. Nevertheless, adsorption isotherm models primarily provide mathematical descriptions of equilibrium behavior. Although they can offer valuable mechanistic indications, model fitting alone cannot definitively identify adsorption mechanisms and should always be interpreted together with kinetic studies, thermodynamic analyses, spectroscopic characterization, and independent evidence regarding adsorbate–adsorbent interactions [80]. It should be emphasized that kinetic models and adsorption isotherms primarily describe experimental behavior and should not be interpreted as direct proof of chemisorption, physisorption, ion exchange, or other specific mechanisms.

#### 3.3.3. Adsorption Thermodynamics

Thermodynamic studies are an essential part of characterizing adsorption processes, as they provide information on the spontaneity of the process, the energy effects that occur during adsorbate binding, and changes in the order of the system. Thermodynamic parameters are most often determined based on adsorption experiments conducted at various temperatures and then analyzed using the van’t Hoff equation [84]. The most important thermodynamic parameters include the change in Gibbs free energy ($∆G^{0}$), the change in enthalpy ($∆H^{0}$) and the change in entropy ($∆{GS}^{0}$). These parameters make it possible to assess whether an adsorption process can proceed spontaneously, whether it is exothermic or endothermic, and the interactions at the adsorbent–solution interface. The standard Gibbs free energy change associated with adsorption is commonly determined from the thermodynamic equilibrium constant according to Equation (1):

(1) $$∆G^{0}=-RT\ln K,$$

where $∆G^{0}$ is the standard Gibbs free energy change [kJ/mol], R is the universal gas constant (0.008314 kJ/molK), T is the absolute temperature [K] and K is a dimensionless thermodynamic equilibrium constant [84].

For adsorption systems, particular care must be taken when defining K because thermodynamic relationships require a dimensionless quantity. Experimental studies frequently report distribution coefficients, Langmuir affinity constants, Freundlich constants or concentration ratios that possess physical units and therefore cannot be directly substituted into Equation (1). When such parameters are used, they should first be converted to a dimensionless equilibrium constant using the corresponding standard-state convention and activity-based formulation described in the thermodynamic literature [85]. In practical adsorption studies, K is often approximated from the adsorption distribution coefficient obtained at equilibrium (Equation (2)):

(2) $$\;K_{d}=\frac{q_{e}}{C_{e}}\;,$$

where $q_{e}$ is the equilibrium adsorption capacity [mg/g] and $C_{e}$ is the equilibrium concentration in solution [mg/L]. However, because $K_{d}$ is dimensional, additional normalization is required before its use in thermodynamic calculations. Therefore, reported values of $∆G^{0}$, $∆H^{0}$, and $∆S^{0}$ should always be interpreted in relation to the equilibrium constant definition adopted by the authors [85].

The values of $∆H^{0}$ and $∆S^{0}$ are commonly obtained from the van’t Hoff equation (3):

(3) $$\ln K=\frac{∆S^{0}}{R}-\frac{∆H^{0}}{RT},$$

where $∆S^{0}$ is the standard entopy change of adsoption [kJ/molK] and $∆H^{0}$ is the standard enthalpy change of adsorption [kJ/mol].

A van’t Hoff plot (Figure 6) is obtained by plotting lnK against $\frac{1}{T}$. The slope and intercept correspond to −$∆H^{0}/R$ and $∆S^{0}/R$, respectively [86].

Negative $∆G^{0}$ values indicate that adsorption is thermodynamically favorable relative to the chosen reference state. However, thermodynamic favorability should not be directly equated with adsorption efficiency in a particular experimental system, because the observed adsorption performance is additionally influenced by factors such as pH, adsorbent dosage, initial concentration, competing ions, and mass-transfer limitations [84].

The change in enthalpy ($∆H^{0}$) provides information about the energetic nature of the adsorption process. Positive $∆H^{0}$ values indicate an endothermic process, in which adsorption is favored at higher temperatures, while negative values indicate an exothermic process. Depending on the adsorbent properties and adsorption mechanism, both endothermic and exothermic adsorption processes have been reported for lignocellulosic biosorbents [87].

The entropy change ($∆S^{0}$) describes variations in the degree of order at the adsorbent–solution interface during adsorption. Positive $∆S^{0}$ values generally indicate increased randomness at the solid–liquid interface, whereas negative values suggest a more ordered system resulting from stronger adsorbent–adsorbate interactions and the formation of more stable surface complexes [86]. 

In recent years, thermodynamic studies have increasingly been combined with kinetic and equilibrium analyses to obtain a more comprehensive description of adsorption mechanisms. The combined interpretation of thermodynamic parameters, adsorption isotherms, and kinetic models provides valuable insights into the feasibility of the process, the nature of adsorbate–adsorbent interactions, and the mechanisms governing adsorption under different operating conditions [84]. This information enables the assessment of their potential for practical application in water and wastewater treatment technologies.

It should be emphasized that the removal of metal ions from aqueous solutions may proceed through several simultaneous mechanisms, including adsorption, ion exchange, surface complexation, precipitation, and redox reactions. Consequently, adsorption isotherms, kinetic models, FTIR spectra, SEM observations, or thermodynamic parameters alone cannot definitively identify the dominant removal mechanism. Reliable mechanistic interpretation generally requires the combined use of multiple complementary analytical techniques and the consideration of solution chemistry, particularly pH-dependent metal speciation and precipitation phenomena. As an example, under certain experimental conditions, especially at elevated pH values, metal removal may be partially associated with precipitation processes rather than adsorption alone.

## 4. Sorption Potential of Selected Biosorbents of Agri-Food Origin

Biosorption is a complex process that can be explained by all sorts of physicochemical interactions that occur between functional groups on the surface of a biosorbent and, for example, metal ions in solution, including complexation, chelation or precipitation [88]. Table 2 provides an overview of the effectiveness of various agri-food-derived biosorbents against selected metal ions in aqueous solutions.

Chand et. al. used apple pomace (AP) as a sorption material for cadmium ions from aqueous solutions [38]. The best-fitting sorption isotherm in this system was the Langmuir isotherm. The maximum sorption capacity (q_max_), achieved by dried and ground material, as calculated, was equal to 4.45 mg Cd(II)/g of AP. The same authors reported a higher maximum adsorption capacity for Pb(II) than for Cd(II) when using apple pomace under the respective experimental conditions investigated in their study [89]. Another pomace material, obtained from olive processing, was tested for its sorption capacity against copper(II) and cadmium(II) ions. Using these, the maximum adsorption capacity was calculated, also using the Langmuir isotherm equation. The sorption capacity for cadmium(II) ions was equal to 3.37 mg/g, tested by Chand and colleagues (4.45 mg/g) [38]. In relation to copper(II) ions, a maximum sorption capacity of 11.44 mg/g was obtained [88].

The adsorption capacity of biomass-derived sorbents cannot be explained solely by the type of precursor material. The abundance of oxygen-containing functional groups, such as hydroxyl (-OH), carboxyl (-COOH), carbonyl (C=O), and phenolic groups, plays a critical role in metal-ion binding through mechanisms including ion exchange, electrostatic attraction, and surface complexation. Consequently, biosorbents with a high concentration of accessible functional groups may exhibit superior adsorption performance even when their specific surface area is relatively low [90]. Differences in adsorption capacity are often associated with variations in surface chemistry rather than solely with textural properties. Therefore, materials with a lower surface area may occasionally outperform thermally treated sorbents if the treatment reduces the abundance of active functional groups.

**Table 2:** Sorption capacity of selected waste products from the agri-food industry.

Sorbent	Adsorbate	Experimental Conditions	Removal Efficiency [%]	Experimental Adsorption Capacity, q_e_ [mg/g]	Maximum Adsorption Capacity, qmax [mg/g]	Isotherm Model	Ref.
Apple pomace	Cd(II)	pH 5–6; sorbent dosage 40 g/L; C_0_ = 40 mg/L	-	-	4.45	Langmuir	[38]
	Pb(II)	pH 4; sorbent dosage 16 g/L; C_0_ = 50 mg/L	-	-	16.39 16.14	Langmuir Freundlich	[89]
Olive pomace	Cu(II)	pH 5; sorbent dosage 10 g/L; C_0_ = 80 mg/L	-	-	11.44	Langmuir	[88]
	Cd(II)	pH 5; sorbent dosage 10 g/L; C_0_ = 50 mg/L	-	-	3.37		
Corn residues mix	Ni(II)	pH 4–5; sorbent dosage 10 g/L; C_0_ = 0.18 mmol/L	58.9	-	-	-	[91]
	Zn(II)	pH 4–5; sorbent dosage 10 g/L; C_0_ = 0.18 mmol/L	82.8	-	-	-	
	Cd(II)	pH 4–5; sorbent dosage 10 g/L; C_0_ = 0.18 mmol/L	90.7	-	-	-	
Wheat shells	Cu(II)	pH 6; sorbent dosage 20 g/L; C_0_ = 500 mg/L	-	11.00	17.42	Langmuir	[92]
Rice shells	Cu(II)	pH 6; sorbent dosage 20 g/L; C_0_ = 500 mg/L	-	3.60	2.95		
Lentil shells	Cu(II)	pH 6; sorbent dosage 20 g/L; C_0_ = 500 mg/L	-	15.62	9.59		
Carrot residues	Cu(II)	pH 5; C_0_ = 20 mg/L	-	-	32.74	Freundlich/ Langmuir	[93]
	Cr(III)	pH 4; C_0_ = 20 mg/L	-	-	45.09		
	Zn(II)	pH 5; C_0_ = 20 mg/L	-	-	29.61		
*Brassica campestris* stems	Pb(II)	pH 4; sorbent dose 20 g/L; C_0_ = 120 mg/L	98.0	-	78.5	Freundlich/ Langmuir	[94]
	Cr(VI)	pH 9; sorbent dose 20 g/L; C_0_ = 120 mg/L	91.0	-	95.0		
	Ni(II)	pH 9; sorbent dose 20 g/L; C_0_ = 120 mg/L	49.0	-	1.1		
Tea waste	Cu(II)	pH 5.5; sorbent dosage 1 g/L; C_0_ = 5 mg/L	-	-	8.64	Freundlich	[95]
	Cd(II)	pH 5.5; sorbent dosage 1 g/L; C_0_ = 5 mg/L	-	-	11.29		
Sunflower seed hulls	Ni(II)	pH 4–5; sorbent dosage 10 g/L; C_0_ = 0.18 mmol/L	71.0	-	-	-	[91]
	Zn(II)	pH 4–5; sorbent dosage 10 g/L; C_0_ = 0.18 mmol/L	89.2	-	-		
	Cd(II)	pH 4–5; sorbent dosage 10 g/L; C_0_ = 0.18 mmol/L	96.6	-	-		

Within the study conducted by Aydın et al. [92], wheat shells exhibited the highest Langmuir-derived adsorption capacity toward Cu(II), whereas lentil shells showed the highest experimentally determined adsorption capacity. The sorption capacity of all of biosorbents tested by these authors [92] increased as the initial sorbate concentration increased from 100 to 500 mg/L. A similar relationship was obtained with the temperature increase. Although computational data, based on the Langmuir isotherm equation, showed that wheat shells had the highest sorption capacity (17.42 mg/g) and lentil shells ranked second among the three sorbents tested (with q_max_ of 9.59 mg/g), experimental data showed that lentil shells achieved a higher sorption capacity value than wheat shells, at 15.62 and 11.00 mg/g, respectively.

From experimental data for Cr(III), Cu(II) and Zn(II) sorption on carrot residues, there was a slightly better fit to the Freundlich sorption isotherm equation than the Langmuir equation. Of the three metal solutions tested, the highest maximum sorption capacity of carrot residue was noted for chromium ions (45.09 mg/g) [93]. In a paper on the sorption performance of washed and sized tea waste against copper(II) and cadmium(II) ions [95], a better fit of the process to the Freundlich adsorption isotherm was also demonstrated. In contrast to the olive pomace study by Acar et al. [88], tea waste exhibited a higher Langmuir/Freundlich-derived adsorption capacity toward Cd(II) than toward Cu(II) under the experimental conditions applied by the authors.

Determination of the sorption activity of biomass residues selected by Simón and colleagues [91] (sunflower seed hulls—SSH and corn) showed that, at a concentration of 0.18 mmol, each of the metal ions analyzed (among Ni(II), Zn(II) and Cd(II)) was removed with an efficiency of at least 54.4%. For each raw material, the highest efficiency was observed in the adsorption of cadmium(II) ions. Nickel ions have a higher hydration energy than the other two ions, which makes it more difficult to remove the water molecules from their sphere and there is a lower probability of their adsorption [91].

The highest metal-ion removal on *Brassica campestris* stems as a biosorbent, achieved by Shaikh and others [94], was for lead(II) ions, with a value of 98%. For chromium(III) and nickel(II) ions, the removal efficiency was equal to 91% and 49%, respectively, using a sorbent dose of 20 g/L. For Cr(VI) and Ni(II) ions, the optimal process pH was 9, but for lead(II) ions it was much lower, about 4.

The studies summarized in Table 2 demonstrate that a wide range of agri-food residues can effectively remove metal ions from aqueous solutions. Reported adsorption capacities varied substantially among sorbents; however, direct comparison between studies should be interpreted with caution because adsorption performance is strongly influenced by experimental variables such as initial metal concentration, sorbent dosage, pH, temperature, and biomass pretreatment procedures. Within the reviewed studies, adsorption capacities ranged from 2.95 mg/g for rice shells toward Cu(II) [92] to 95.0 mg/g for Cr(VI) adsorption on *Brassica campestris* stems [94]. For Cd(II) adsorption, reported maximum capacities varied between 3.37 mg/g for olive pomace [88] and 11.29 mg/g for tea waste [95], while Cd(II) removal efficiencies reached up to 96.6% for sunflower seed hulls [91]. Most studies reported optimal adsorption in the pH range of 4–6, although adsorption of Cr(VI) and Ni(II) on *B. campestris* stems was optimized at pH 9 [93].

## 5. Sorption Potential of Selected Biosorbents of Agri-Food Origin, Subjected to Modifications

In general, functional groups that favor adsorption are oxygen-containing, depending on the type of adsorbent; hydroxyl, carboxyl, sulfate, phosphate, and amine groups are among the most common [96]. Their contribution to adsorption is primarily related to their ability to bind metal ions through complexation and ion-exchange mechanisms. The ionization state of these groups depends strongly on pH. For example, carboxyl groups are predominantly protonated (-COOH) at low pH, whereas increasing pH promotes their dissociation to negatively charged carboxylate groups (-COO^−^). Consequently, adsorption of metal cations through ion exchange and surface complexation is generally favored when a sufficient fraction of these functional groups is present in the dissociated form. An increasing number of chemical and thermal biomass-modification strategies have been investigated to increase the abundance of functional groups capable of complexation or ion exchange [97,98,99,100]. Crucial to the optimization of the adsorption process is the identification and quantification of the active sites responsible for contaminant binding. These characteristics are closely related to the modification methodology applied, including the chemicals used and the treatment temperature [101]. Table 3 provides an overview of chemically/thermally modified agri-food-derived adsorbents in terms of their sorption activity against metal ions [38].

### 5.1. Chemical Modifications

Apple pomace (AP) is a raw material rich in polyphenol compounds, characterized by the presence of reactive hydroxyl groups [38]. Modification by replacing hydrogen in their molecules with compounds that chelate metal ions better can increase the sorption capacity of this biosorbent. Such compounds include esters, xanthates, thiols and others [109,110,111]. They affect not only the sorbent capacity but also its specificity towards different sorbates. Modification of AP with succinic anhydride resulted in the achievement of a biosorbent surface area 18% greater than that of raw AP. Chemically modified AP achieved a higher sorption capacity towards cadmium(II) ions than unmodified AP, with maximum sorption capacities of 91.75 and 4.45 mg/g, respectively [38]. A similar team of researchers also modified AP with the goal of increasing its sorption activity against Pb(II), Cd(II) and Ni(II) ions. They used chemical modification by synthesizing methyl acrylate on the surface of AP [102], resulting in the formation of polymerized AP. Compared to unmodified AP, the maximum sorption efficiency for all three metal ions increased with the use of polymerized AP, which also required less sorbent mass to effectively remove these ions from aqueous solutions [38,89]. The highest degree of removal of ions of three metals was observed in the 10–80 mg/L concentration range. On the basis of the Langmuir isotherm model, the maximum sorption capacities against three metal ions were calculated. They were equal to 106, 34.12 and 19.45 mg/g for sorption of Pb(II), Cd(II) and Ni(II) ions, respectively. In addition, a sorption kinetics analysis was performed using a pseudo-second-order reaction model. Moreover, analysis of thermodynamic parameters showed that the adsorption of lead and cadmium ions was an endothermic reaction [38].

The adsorption isotherm equation for olive pomace (OP) modified with H_2_O_2_ and H_3_PO_4_ for Cu(II) and Cd(II) sorption was again a better match for the Langmuir isotherm [88]. Comparing the sorption capacities of unmodified (Table 2) H_2_O_2_-treated and H_3_PO_4_-treated OP, a higher degree of copper ion removal was noted than of cadmium ion removal. The maximum sorption capacity against Cu(II) and Cd(II) ions was calculated for acid-treated pomace, yielding values of 31.77 mg for copper ions and 11.20 mg for cadmium ions per gram of sorbent, respectively. Hydrogen peroxide treatment resulted in a slight increase in sorbent yield, achieving an increase in Cu(II) adsorption capacity from 11.44 to 12.07 mg/g and a greater increase in Cd(II) adsorption capacity from 3.37 to 5.62 mg/g [88]. The observed differences in adsorption behavior cannot be explained solely by the metal-ion mass. Instead, they are more likely related to differences in metal speciation, hydration characteristics, and affinity toward the functional groups present on the sorbent surface. In particular, interactions between metal ions and oxygen-containing functional groups may differ depending on the stability of the complexes formed and the extent of ion hydration, both of which can influence adsorption efficiency [112,113].

The adsorption capacity of groundnut hulls has so far been proven against copper(II) and lead(II) ions [114]. Owalude and Tella [106] focused their work on the sorption of chromium(VI) ions on this adsorbent, both unmodified and chemically modified with pyromellitic dianhydride (PMDA), with the ability to enrich the adsorbent’s surface with carboxyl groupings. Images of the sorbent before and after sorption of chromium (VI) ions showed the disappearance of pores that were visible on the surface of the sorbent before sorption. It was deduced that adsorption of these ions had occurred. FT-IR spectra of unmodified and PMDA-modified sorbent before and after sorption of Cr(VI) ions provided more precise information about which functional groups were involved in the adsorption process. First, a signal from a carbonyl group appeared on the PMDA-modified sorbent spectrum, confirming its correct synthesis. Second, after the sorption process, peaks from hydroxyl and carbonyl groups flattened, confirming their participation in the binding of Cr(VI) ions. By bringing a pH of a chromium(VI) solution ions to 2, the highest degree of chromium(VI) ion removal (about 96%) was achieved using PMDA-modified sorbent. Under the same conditions, unmodified groundnut shells achieved removal efficiency of 82%. It was concluded that a positive charge in the sorbent surface with H^+^ ions plays an essential role, since negatively charged chromate ions present in solution can easily, by electrical attraction, be bound to functional groups of the sorbent surface. This example also illustrates that adsorption behavior depends not only on contaminant concentration but also on aqueous speciation. In contrast to divalent metal cations, Cr(VI) and arsenic species are commonly present as oxyanions under environmentally relevant pH conditions, resulting in different adsorption mechanisms and pH optima [113]. Based on the Langmuir isotherm equation, the maximum adsorption capacity was calculated. For a sorbent without any chemical modification, the figure was 90 mg/g, while for a modified sorbent it was equal to 131 mg/g. At higher Cr(VI) concentrations, unmodified groundnut shells were more effective, but a PMDA-modified adsorbent proved more effective under the most variable conditions [106]. Comparing the sorption capacity of *B. campestris* stems against Cr(VI) ions [94], raw groundnut shells were similarly capable of removing these ions from solutions. Indeed, the maximum sorption capacities of these two sorbents were 95 and 90 mg/g for *B. campestris* stems and raw groundnut shells, respectively. However, modified groundnut shells were more efficient.

Chand and Pakade [59] pointed out the high sorption capacity of hydroxyapatite nanoparticles. Advantages in favor of its use include its low degradability in water and synthesis cost. By synthesizing it on a biomass matrix of AP, the authors designed the most effective sorbent for Pb(II), Cd(II) and Ni(II) ions based on this raw material among those included in this review. Studies of the surface of AP before and after modification provided information on the increase in surface heterogeneity during impregnation with hydroxyapatite nanoparticles. This was evidenced by an increase in particle size and a decrease in zeta potential after the sorption processes. The most optimal pH of sorbate solutions was around 5. The sorption efficiency was compared depending on the dose of apple pomace used to remove cadmium and lead ions (Table 2) [38,89]. One of the positive results of the study is that a smaller sorbent weight was required for the sorption of metal ions using nanohydroxyapatite-modified AP compared to the use of unmodified pomace, with much higher sorption capacities of 303 mg/g and 250 mg/g for hydroxyapatite-modified pomace, compared with 16.39 mg/g and 4.45 mg/g for raw AP, for Pb(II) and Cd(II), respectively. The sorption of nickel(II) ions was less effective, with a maximum sorption capacity equal to 100 mg/g, using nano-modified AP [59]. For AP modified with methyl acrylate, the sorption capacity was lower, with maximum sorption capacities of 106, 34.12 and 19.45 mg/g for Pb(II), Cd(II) and Ni(II) ions, respectively, at a slightly lower pH of 4 [89,102]. The study of sorption kinetics provided further information on the benefits of using nano-hydroxyapatite AP, i.e., the very rapid sorption of metal ions on the sorbent, probably by ion exchange with calcium ions in a hydroxyapatite structure. Langmuir proved to be the best adsorption fit. The authors also studied the effect of the co-occurrence of metal ions in solution on their removal. They analyzed the effect of metal ions on each other in terms of their sorption efficiency in binary systems: Pb(II) and Cd(II), Pb(II) and Ni(II), and Cd(II) and Ni(II). The results of the study show that the sorption efficiency of nickel(II) ions was negatively affected by the presence of both cadmium(II) and lead(II) ions. The sorption of Cd(II) ions was slightly affected by the presence of Pb(II) and Ni(II), whereas Pb(II) adsorption remained essentially unchanged in the presence of competing ions. This behavior may indicate a stronger affinity of Pb(II) for the available adsorption sites. Such selectivity can be influenced by differences in metal-ion speciation, hydration energy, hydrated ionic radius, and the stability of surface complexes formed with the functional groups of the adsorbent. Therefore, competitive adsorption is likely governed by a combination of physicochemical factors rather than simple differences in atomic mass [112,115]. This is also evidenced by the coefficient b value, calculated from the Langmuir isotherm, which indicates the ability of a metal ion to bind to the surface of the sorbent. In the case of lead(II) ions, its value was higher than for cadmium(II) and nickel(II) ions. Furthermore, at alkaline pH values, metal removal may result not only from adsorption but also from the precipitation of metal hydroxides. Therefore, adsorption capacities determined under such conditions should be interpreted with caution, and the potential contribution of precipitation should be considered when discussing adsorption mechanisms [116].

Manzoor and colleagues [105] investigated the sorption efficiency for chromium(III) ions using corn cob waste biomass, which was immobilized in a calcium alginate matrix. It was shown that for this sorbent, the most favorable pH for removal of Cr(III) ions was 5, at which the obtained maximum sorption capacity was 277.57 mg/g. Moreover, the efficiency of Cr(III) ion removal from tannery wastewater was tested. Using immobilized corn cob waste, almost 65% of Cr(III) ions were removed.

### 5.2. Other Modifications

Zama and colleagues [104] tested biochar plant materials as sorbents for Pb(II) and Cd(II) in aqueous solutions. For each raw material (peanut shells—PS, and buckwheat husk—BH), a pyrolysis process was performed at different temperatures: 350, 450, 550 and 650 °C. The effect of the type of sorbent and temperatures on their maximum sorption capacity was then compared. In the case of Pb(II) ions, which were the most susceptible to sorption reactions, an increase in the temperature at which the biochar was obtained resulted in a slight increase in the degree of metal-ion removal, e.g., for BH there was an increase from 99.7% to 99.8%. Cadmium(II) ions were most effectively removed by biochar obtained at 350 °C (PS) and 450 °C (BH). Sorption of cadmium(II) ions yielded no clear evidence of complexation reactions, but only of precipitation reactions with mineral anions bound to the biochar surface and ion exchange. Using the sorbents tested, a maximum 99.2% of Cd(II) ions was removed using PS biochar, while BH biochar removed 60.8%. The Freundlich isotherm was a better fit to the lead(II) ion sorption data, indicating heterogeneous multilayer sorption. Cadmium(II) ions were adsorbed according to the Langmuir isotherm equation on BH, while, when using PS, the Freundlich equation was a better fit.

Another lead(II) removal biochar-based biosorbent was sugarcane bagasse [103]. To enhance the sorption properties, the authors conducted an anaerobic digestion process of the biochar, using a thermophilic digester at 55 °C. Then, digested and raw biomass was pyrolyzed at 600 °C to achieve two different adsorbents, which were then tested for sorption capacities in comparison to commercially used activated carbon. From the BET surface areas, it was concluded that the activated carbon, with a larger surface area (1100 m^2^/g), could have a higher sorption capacity than the studied biochar, with values lower than 20 m^2^/g. However, the batch sorption results yielded the opposite conclusion, that is, the highest maximum sorption capacity was obtained for digested biochar. All the isotherms were well described by the Langmuir model, from which it was deduced that digested sugarcane bagasse biochar had a higer bonding ability for lead(II) ions than for other, non-digested bagasse biochar and activated carbon. This resulted in the highest sorption capacity of the material, reaching a maximum value of 135.49 mg/g, which was approximately 1.65 times higher than that of commercial activated carbon (81.90 mg/g) and more than 20 times higher than that of raw sugarcane bagasse biochar (6.49 mg/g). An important advantage of this approach is its lower cost compared with techniques requiring the addition of various chemicals, while still providing a substantial improvement in adsorption performance. In addition, the authors evaluated the sorption capacity of commercial activated carbon under the same experimental conditions. Their results indicated that anaerobically digested sugarcane bagasse biochar exhibited a sorption capacity approximately 1.65 times higher than commercial activated carbon and more than 20 times higher than untreated sugarcane bagasse biochar.

Table 3 summarizes the adsorption capacities reported for modified biomass-based sorbents investigated in the cited studies. Because the adsorption performance was determined under different experimental conditions, including initial metal concentration, pH, contact time, adsorbent dosage, and data-analysis approaches, direct quantitative comparisons between sorbents from different studies should be interpreted with caution. Nevertheless, the reviewed studies demonstrate that various modification strategies can substantially improve the sorption performance of plant-derived materials toward inorganic pollutants. In the study of hydroxyapatite-impregnated apple pomace (AP), high adsorption capacities were reported for Cd(II), Pb(II), and Ni(II) ions, indicating the beneficial effect of hydroxyapatite incorporation on metal uptake [59]. For Cu(II) removal, thermally treated oil-tea camellia shells modified with MnAl-layered double hydroxides showed a high adsorption capacity in the reported experiments [107]. Buckwheat husk biochar was successfully applied for As(III) adsorption from aqueous solutions [104]. The studies on chromium removal demonstrated promising adsorption capacities for both immobilized corncob activated carbon toward Cr(III) [105] and PMDA-modified groundnut hull toward Cr(VI) [106]. Overall, the collected literature data indicate that chemical and mineral-based modifications of plant biomass can significantly enhance the adsorption properties of biosorbents; however, differences in experimental methodologies limit direct comparison of adsorption capacities reported in separate studies.

### 5.3. Influence of Biomass Modification on Adsorption Performance

The adsorption performance of biomass-derived sorbents is strongly influenced by the degree of material processing. Raw agricultural residues can often be used directly after drying and milling, offering low cost and minimal environmental impact. However, their adsorption capacity may be limited by a low surface area and low number of adsorption sites. Therefore, various modification strategies have been developed to enhance sorption performance. Chemical treatment using acids or oxidizing agents may introduce additional functional groups and increase the availability of active binding sites. Thermal treatments (e.g., torrefaction, pyrolysis) can improve pore development. More advanced materials such as activated carbons typically exhibit higher specific surface areas and enhanced adsorption capacities, although their production requires additional energy and processing steps. Consequently, the selection of a biosorbent should consider not only adsorption capacity but also production costs, energy requirements, reagent consumption and environmental sustainability. In some practical applications, moderately efficient low-cost biosorbents may represent a more sustainable alternative than highly engineered sorbents. Table 4 summarizes the main characteristics of raw and modified biomass, highlighting their adsorption performance, processing requirements, economic aspects and environmental implications. The qualitative rankings presented in Table 4 should be interpreted as indicative trends rather than universal classifications. Actual cost and environmental burdens vary substantially depending on biomass source, transportation distance, drying requirements, modification chemistry, energy source, process scale and regeneration strategy. Consequently, life-cycle assessment (LCA) and techno-economic analysis (TEA) are recommended before generalizing environmental or economic advantages of a particular biosorbent preparation route.

## 6. Decision Framework for Selecting Agri-Food-Derived Adsorbents

**Figure 7:**
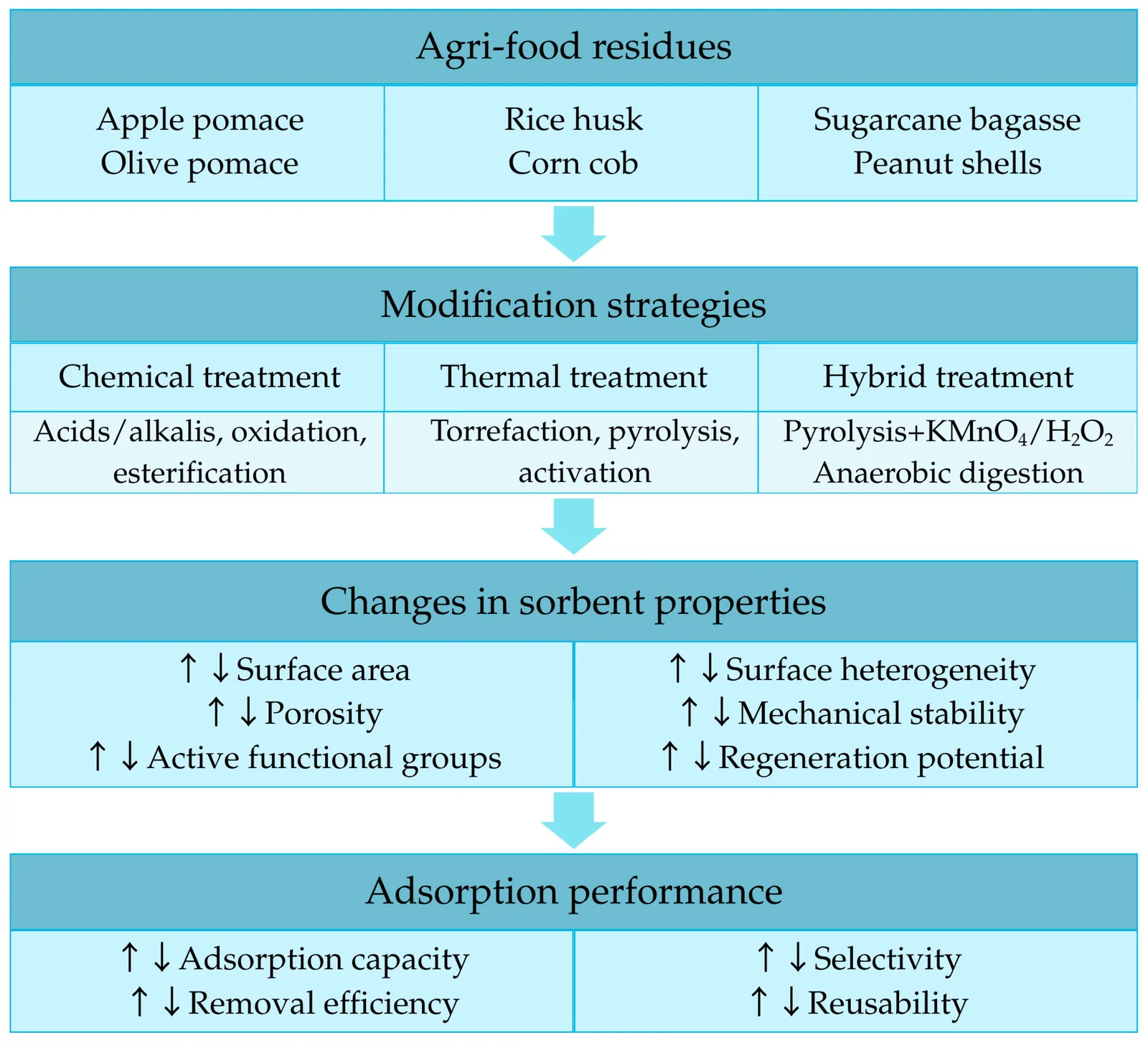
Conceptual framework illustrating potential relationships between agri-food residue feedstocks, modification strategies and performance indicators relevant to water-treatment applications. Depending on the biomass type and modification method, modifications can increase, decrease or have negligible effects on different performance indicators (own work).

The selection of suitable agri-food-derived adsorbents for practical water-treatment applications requires the consideration of multiple interconnected factors. Adsorption performance is influenced not only by the type of biomass feedstock but also by the modification strategy employed, the resulting physicochemical properties of the material, and the dominant adsorption mechanisms involved. These factors collectively determine the adsorption capacity, selectivity, regeneration potential, and, ultimately, the feasibility of scale-up and practical implementation. The relationships among feedstock characteristics, modification approaches, sorbent properties, and performance indicators are summarized in Figure 7. It should be noted that biomass modification does not universally improve all performance indicators. Depending on the feedstock, treatment conditions and target contaminant, modification may improve selected properties while leaving others unchanged or even adversely affecting properties such as regeneration efficiency, mechanical stability or production cost.

Based on the relationships summarized in Figure 7 and the literature analyzed in this review, a practical decision framework (Table 5) is proposed to facilitate biosorbent selection and translation from laboratory studies to water-treatment applications. Unlike previous reviews, which primarily catalog adsorption capacities, the framework integrates adsorption mechanisms, availability of biomass feedstocks, modification requirements, and scalability considerations.

Firstly, priority should be given to residues generated in large quantities and available within existing agricultural or food-processing chains. The first criterion, feedstock availability, determines whether a material can be sourced in sufficient quantities for continuous operation. Apple pomace, grape pomace, citrus peels, olive pomace, and sugarcane bagasse are particularly attractive because their continuous generation can support industrial-scale sorbent production. In contrast, niche residues or seasonally limited wastes may be valuable for laboratory studies but are less suitable for large-scale deployment. Moreover, adsorbents should be selected according to the dominant removal mechanism: ion exchange, surface complexation, electrostatic attraction, precipitation and more. Understanding these mechanisms is essential because adsorption capacity alone does not predict performance under complex water matrices. Also, results obtained in single-metal systems should be interpreted with caution because competing ions frequently reduce adsorption performance. Studies incorporating binary or multi-component systems provide more realistic estimates of adsorbent selectivity and operational effectiveness. Furthermore, adsorbent modifications should be justified by a substantial gain in adsorption capacity, selectivity, or regeneration potential. Simple oxidative treatments may provide moderate improvements, whereas hydroxyapatite impregnation or LDH-based composites often provide order-of-magnitude increases in adsorption capacity but at a higher processing cost. Additionally, adsorbents showing excellent batch performance should not automatically be considered suitable for industrial implementation. The next factor, scalability, determines whether a feedstock can be efficiently processed into a mechanically stable and regenerable sorbent suitable for fixed-bed column operation. Materials that are easy to dry, grind, pelletize, or incorporate into composite structures (such as sugarcane bagasse, rice husks, and nut shells) offer clear advantages in terms of mechanical durability, hydraulic performance, and compatibility with industrial sorption systems. Scalability therefore integrates processing cost, mechanical stability, regeneration potential, and the feasibility of transitioning from batch experiments to continuous-flow systems. Additional criteria should include, but are not limited to: hydrolytic stability, pressure-drop performance, regeneration efficiency, breakthrough characteristics, and resistance to particle degradation. Therefore, fixed-bed column studies should be considered a necessary intermediate step before real-world deployment.

Although agri-food waste-derived adsorbents are frequently described as low-cost materials, their overall economic feasibility depends on several processing and operational factors. Most biosorbents require drying, grinding, sieving, and storage prior to use, generating additional energy demand and operating costs. Furthermore, chemically modified materials often require acids, alkalis, oxidizing agents, or mineral precursors, increasing material consumption and waste generation. The economic benefit associated with the use of agricultural residues therefore cannot be assessed solely on the basis of feedstock availability. The costs of preparation, modification, regeneration, transportation, and final disposal must also be considered. Furthermore, regeneration is a critical factor influencing process sustainability. While numerous laboratory studies report successful desorption of metal ions using acidic or chelating solutions, adsorption capacity frequently decreases over successive cycles. Moreover, repeated regeneration may alter surface functional groups, reduce mechanical stability, and lead to sorbent degradation [7,52]. Another important consideration is the environmental stability of modified agri-food-derived adsorbents. The potential release of adsorbed metals or components introduced during sorbent modification should be evaluated during regeneration and end-of-life management, as these aspects remain insufficiently addressed in many laboratory-scale studies [7]. Consequently, elution assessments should accompany adsorption studies before practical application can be recommended. End-of-life management remains insufficiently addressed in the literature. Metal-loaded adsorbents may require stabilization, recovery of adsorbed metals, thermal treatment, or disposal as hazardous waste depending on the metal concentration and regulatory requirements.

To illustrate the practical use of the proposed framework, hydroxyapatite-modified apple pomace may be considered as an example candidate material for Pb(II) removal. Apple pomace fulfills the feedstock-availability criterion because it is generated in large quantities by the juice and cider industry [14,119]. The material also exhibits high adsorption performance, with a reported Langmuir capacity of 303 mg/g toward Pb(II) [59]. Furthermore, competitive adsorption experiments demonstrated relatively high selectivity for Pb(II) compared with Cd(II) and Ni(II) [59]. However, application of the framework also reveals several evidence gaps. Although adsorption performance has been demonstrated, limited information is available regarding regeneration efficiency over multiple cycles, long-term mechanical stability, pressure-drop behavior in fixed-bed systems, and full economic assessment of hydroxyapatite impregnation. According to the criteria proposed in Table 1 and Table 5, these missing data prevent a complete assessment of industrial applicability despite the excellent batch adsorption performance. Consequently, the framework would classify hydroxyapatite-modified apple pomace as a highly promising candidate requiring further validation through regeneration studies, fixed-bed column experiments, and economic assessment before large-scale implementation can be recommended.

## 7. Conclusions

This review demonstrates that agri-food-derived biosorbents constitute a technically promising approach for the removal of heavy metals from aqueous systems. Across the reviewed literature, chemically and thermally modified biosorbents generally exhibited higher adsorption capacities than their unmodified counterparts, with hydroxyapatite-impregnated apple pomace representing one of the most effective materials reported for Pb(II), Cd(II), and Ni(II) adsorption under laboratory conditions. These findings highlight the importance of surface chemistry, pore structure, and material modification in determining adsorption performance. At the same time, currently available evidence is largely derived from batch-scale experiments and cannot be directly interpreted as proof of industrial feasibility.

It should be emphasized that, in practice, economic considerations will primarily determine which raw material will be used as a biosorbent. Therefore, particular attention should be given to waste streams generated in large quantities, as these materials offer the greatest potential for industrial implementation. One such example is apple pomace, which is one of the most abundant by-products of the fruit-processing industry. During juice and cider production, approximately 25–35% of the processed apple mass remains as pomace, creating a substantial biomass stream with significant potential for valorization and environmental applications [120]. Studies have shown that both chemical and biological methods of modifying biosorbents make it possible to improve their properties. The pyrolysis process described in the paper is one example that is of great interest to scientists and industry.

The increasing level of water and wastewater contamination creates a strong need for efficient and sustainable treatment technologies. Agri-food-derived biosorbents represent promising candidates owing to their availability, renewability, and favorable adsorption properties. Nevertheless, industrial applicability should currently be regarded as prospective rather than demonstrated. While several biomass-derived materials have shown encouraging fixed-bed column performance and excellent adsorption capacities in laboratory studies, comprehensive evidence regarding large-scale operation, regeneration costs, adsorbent lifetime, hydraulic performance, and treatment economics remains limited. Future research should therefore move beyond adsorption capacity comparisons and include fixed-bed operation, breakthrough analysis, regeneration studies, life-cycle assessment, and techno-economic evaluation. In particular, treatment costs should be evaluated on the basis of the volume of purified water or the mass of contaminant removed rather than solely the cost per kilogram of sorbent. Such information will be essential for determining whether biosorbent-based technologies can compete with established water-treatment processes under real operating conditions.

## Figures and Tables

**Table 1:** System for evaluating biosorbents for batch experiments scale-up.

Stage	Key Question	Proposed Indicator
Batch adsorption	Does the material exhibit sufficient affinity?	Maximum adsorption capacity, removal efficiency
Regeneration	Can the material be reused?	Capacity retention after cycles [%]
Elution assessment	Are modifying agents stable?	Leaching tests
Column operation	How rapidly does breakthrough occur?	Breakthrough time
Dynamic performance	How much capacity is usable under flow conditions?	Usable bed capacity
Mechanical stability	Does the sorbent maintain structural integrity?	Degradation, pressure-drop resistance
Scale-up potential	Can the process operate continuously?	Fixed-bed performance

**Table 3:** Sorption capacity of modified waste products from agri-food industry.

Sorbent	Adsorbate	Experimental Conditions	Removal Efficiency [%]	Experimental Adsorption Capacity, q_e_ [mg/g]	Maximum Adsorption Capacity, q_max_ [mg/g]	Isotherm Model	Ref.
Apple pomace treated with succinic anhydride	Cd(II)	pH 5–6; sorbent dosage 0.8 g/L; C_0_ = 50 mg/L	-	-	91.75	Langmuir	[38]
Methyl acrylate modified apple pomace	Pb(II)	pH 4; sorbent dosage 4 g/L; C_0_ = 80 mg/L	-	-	106.0	Langmuir	[102]
	Cd(II)	pH 4; sorbent dosage 4 g/L; C_0_ = 80 mg/L	-	-	34.12	Langmuir	
	Ni(II)	pH 4; sorbent dosage 4 g/L; C_0_ = 80 mg/L	-	-	19.45	Langmuir	
Olive pomace treated with H_2_O_2_	Cu(II)	pH 5; sorbent dosage 10 g/L; C_0_ = 80 mg/L	-	-	12.07	Langmuir	[88]
	Cd(II)	pH 5; sorbent dosage 10 g/L; C_0_ = 50 mg/L	-	-	5.62	Langmuir	
Olive pomace treated with H_3_PO_4_	Cu(II)	pH 5; sorbent dosage 10 g/L; C_0_ = 80 mg/L	-	-	31.77	Langmuir	
	Cd(II)	pH 5; sorbent dosage 10 g/L; C_0_ = 50 mg/L	-	-	11.20	Langmuir	
Hydroxyapatite nanoparticles impregnated on apple pomace	Pb(II)	pH 5; sorbent dosage 0.4 g/L; C_0_ = 100 mg/L	92.8	-	303	Langmuir	[59]
	Cd(II)	pH 5; sorbent dosage 0.8 g/L; C_0_ = 100 mg/L	100	-	250	Langmuir	
	Ni(II)	pH 5; sorbent dosage 1.2 g/L; C_0_ = 80 mg/L	68.9	-	100	Langmuir	
Anaerobically digested sugarcane bagasse biochar	Pb(II)	sorbent dosage 2 g/L; C_0_ = 20 mg/L	-	-	135.49	Langmuir	[103]
Peanut shells biochar	Pb(II)	pH 5.5; sorbent dosage 5 g/L; C_0_ = 5 mg/L	99.7	-	-	Freundlich	[104]
	Cd(II)	pH 5.5; sorbent dosage 5 g/L; C_0_ = 5 mg/L	99.2	-	-	Freundlich	
Buckwheat husk biochar	Pb(II)	pH 5.5; sorbent dosage 5 g/L; C_0_ = 5 mg/L	99.8	-	-	Freundlich	
	Cd(II)	pH 5.5; sorbent dosage 5 g/L; C_0_ = 5 mg/L	60.8	-	-	Langmuir	
Immobilized corncob activated carbon	Cr(III)	pH 5; sorbent dosage 1 g/L; C_0_ = 1000 mg/L	27.76	277.57	-	Langmuir	[105]
Pyromellitic dianhydride (PMDA) modified groundnut hull	Cr(VI)	pH 2; sorbent dosage 0.4 g/L; C_0_ = 12.5 mg/L	-	-	131	Langmuir	[106]
Sugarcane bagasse biochar	Pb(II)	sorbent dosage 2 g/L; C_0_ = 20 mg/L	-	-	6.49	Langmuir	[103]
Oil-tea camellia shells biochar	Cu(II)	pH 3.5–5; sorbent dosage 3.2 g/L	-	20	74.07	Langmuir	[107]
Commercial activated carbon	Cu(II)	pH 5; C_0_ = 25 mg/L	-	5.1	-	Langmuir	[108]
	Ni(II)	pH 8; C_0_ = 25 mg/L	-	2.9	-	Langmuir	
	Pb(II)	sorbent dosage 2 g/L; C_0_ = 20 mg/L	-	-	81.9	Langmuir	[103]

**Table 4:** Comparison of biomass processing strategies (own work based on [6,90,117,118,119]).

Sorbent Type	Processing Level	Surface Area	Adsorption Capacity	Cost	Environmental Footprint
Raw biomass	Low *	Low/Medium	Low/Medium	Very low	Very low
Chemically modified biomass	Medium	Medium	Medium/High	Medium	Medium
Torrefied biomass	Medium	Medium	Medium/High	Medium	Medium
Biochar	High	High	High	Medium	Medium
Activated carbon	Very high	Very high	Very high	High	High

* The ratings presented in this table (low, medium, high) are qualitative and represent general trends reported in the literature. Actual values may vary depending on the biomass type, modification method, operating conditions, and target metal ion.

**Table 5:** Proposed decision framework for selecting agri-food-derived adsorbents.

Criterion	Key Question	Recommended Indicator
Feedstock availability	Is the residue generated in large quantities?	Annual production, local accessibility
Surface chemistry	Which functional groups dominate?	FTIR, elemental analysis
Physical form and handling	Is the sorbent suitable for continuous operation?	Pelletization, particle size, attrition resistance
Adsorption mechanism	Which processes control removal?	Isotherm and mechanism studies
Multi-ion performance	How sensitive is adsorption to competing ions?	Binary/multi-component experiments
Modification benefit	Does modification significantly improve performance?	Maximum adsorption capacity, regeneration ability
Scale-up potential	Can the material operate in a column?	Breakthrough studies
Dynamic column validation	Does the material retain performance under flow conditions?	Thomas, Yoon–Nelson and BDST models, breakthrough studies
Economic feasibility	Is treatment cost justified?	Cost per m^3^ treated water, cost per kg contaminant removed; preparation and regeneration costs
Circularity	Can the sorbent be regenerated or valorized after use?	Regeneration cycles

## Data Availability

No new data were created or analyzed in this study. Data sharing is not applicable to this article.
